# How Does Response Inhibition Influence Decision Making When Gambling?

**DOI:** 10.1037/xap0000039

**Published:** 2015-01-05

**Authors:** Tobias Stevens, Damien Brevers, Christopher D. Chambers, Aureliu Lavric, Ian P. L. McLaren, Myriam Mertens, Xavier Noël, Frederick Verbruggen

**Affiliations:** 1 School of Psychology, University of Exeter; 2 Laboratoire de Psychologie Médicale et d’Addictologie, Université Libre de Bruxelles; 3 School of Psychology, Cardiff University; 4 School of Psychology, University of Exeter; 5 Laboratoire de Psychologie Médicale et d’Addictologie, Université Libre de Bruxelles; 6 School of Psychology, University of Exeter

**Keywords:** executive control, response inhibition, gambling, risk taking

## Abstract

Recent research suggests that response inhibition training can alter impulsive and compulsive behavior. When stop signals are introduced in a gambling task, people not only become more cautious when executing their choice responses, they also prefer lower bets when gambling. Here, we examined how stopping motor responses influences gambling. Experiment 1 showed that the reduced betting in stop-signal blocks was not caused by changes in information sampling styles or changes in arousal. In Experiments 2a and 2b, people preferred lower bets when they occasionally had to stop their response in a secondary decision-making task but not when they were instructed to respond as accurately as possible. Experiment 3 showed that merely introducing trials on which subjects could not gamble did not influence gambling preferences. Experiment 4 demonstrated that the effect of stopping on gambling generalized to different populations. Further, 2 combined analyses suggested that the effect of stopping on gambling preferences was reliable but small. Finally, Experiment 5 showed that the effect of stopping on gambling generalized to a different task. On the basis of our findings and earlier research, we propose that the presence of stop signals influences gambling by reducing approach behavior and altering the motivational value of the gambling outcome.

Many theorists assume that decision making involves an interplay between automatic and control processes (e.g., Kahneman, 2003; Norman & Shallice, 1986). Automatic processes are considered to be fast, associative, effortless, and easily triggered by information in the environment. In contrast, top-down executive control processes are considered to be slower, more effortful, and goal dependent. Executive processes involve organizing, monitoring, biasing, and altering the settings of lower level cognitive processes such as stimulus detection, response selection, and motor programming (e.g., Verbruggen, McLaren, & Chambers, 2014). This allows us to ignore distracting information in the environment, overcome habits or suppress actions, and adjust decision-making strategies when outcomes are suboptimal (Logan & Gordon, 2001; Miller & Cohen, 2001; Monsell & Driver, 2000; Norman & Shallice, 1986). When the executive control system is otherwise engaged or impaired, automatic processes are thought to guide behavior. For example, patients with lesions to the frontal cortex, which is critical for executive control processes, often become impulsive, take more risks, struggle to overcome temptations, fail to correct errors, and show habitual behavior when it is contextually inappropriate (Duncan, 1986; Milner, 1963; Perret, 1974; Shallice, 1982). Brain stimulation of these brain areas induces similar behaviors in healthy subjects (e.g., Chambers et al., 2006; Knoch et al., 2006; Rushworth, Hadland, Paus, & Sipila, 2002; Verbruggen, Aron, Stevens, & Chambers, 2010).

In a recent study, we used a concurrent load technique to examine how manipulations of executive control influence monetary decisions when gambling (Verbruggen, Adams, & Chambers, 2012). The concurrent load technique is often used to measure the relative contributions of automatic and executive control processes in a task (e.g., Logan, 1979; Kahneman, 2003). The central assumption is that tasks that require control processes tend to compete with each other and that this results in a performance decrement. In contrast, automatic processes are assumed to occur in parallel, so concurrent load influences them less. We used a load manipulation in a novel gambling task that measured decision making under uncertainty. On every trial, subjects were presented with six choice options, which were represented by six adjacent bars (see Figure 1). Each option (or bar) was associated with a certain amount subjects could win; however, they were informed that the higher the amount, the less probable a win. Thus, selecting higher amounts represented “risky bets,” whereas selecting lower amounts represented “safe bets.”^1^ After 3.5 s, the bars started rising, and subjects had to respond when the bars reached a top line (see Figure 1). Healthy young adults performed this task throughout the session. In some blocks (*load* blocks), subjects also had to perform a secondary task. The nature of this task depended on the group to which the subjects were assigned. In the first group (*double-response* group), the secondary task required subjects to execute an additional response when the top of the bars turned black (the double-response signal). In the second group (*stop* group), subjects had to stop the planned choice response when the top of the bars turned black (the stop signal). The signals occurred on one-third of the trials of the load blocks. Monitoring for occasional signals, keeping extra task rules in working memory, and preparing to change action plans (i.e., adding an extra response or withholding the planned response) increases cognitive load (e.g., Vandierendonck, De Vooght, & Van der Goten, 1998; Verbruggen & Logan, 2009). We predicted that decision making would become less regulated in load blocks because of the increased demand for executive control under these conditions (Logan, 1979; Kahneman, 2003; Pashler, 1998). Indeed, we found that subjects in the double-response group tended to place higher bets with a lower probability of winning in load blocks in which double-response signals could occur than in no-load blocks (in which no signals could occur), although this effect failed to reach significance. In contrast, subjects in the stop group placed lower bets with a higher probability of winning in load blocks in which stop signals could occur than in no-load blocks (in which no signals could occur). This effect was statistically significant, as was the Block Type × Group interaction. Thus, different types of cognitive load influenced decision making differently. Follow-up tests indicated that the load effect was not a result of differences in probability learning, block order, or estimation of expected value.[Fig-anchor fig1]

We attributed the effect of a stop load to “a transfer of cautiousness” brought about by having to withhold a response in this condition. Several studies have demonstrated that dealing with stop signals makes people more cautious in executing motor responses (Jahfari, Stinear, Claffey, Verbruggen, & Aron, 2010; Liddle et al., 2009; Lo, Boucher, Paré, Schall, & Wang, 2009; Verbruggen & Logan, 2009; Zandbelt, Bloemendaal, Neggers, Kahn, & Vink, 2013). In Verbruggen et al. (2012), people also became more cautious when executing their choice responses (as indexed by longer choice latencies), and we hypothesized that this stopping-induced motor cautiousness transferred to monetary choice in our gambling task (counteracting the effect of multitask interference observed in the double-response group). This transfer effect could have important practical implications for the treatment of disorders that have been linked to poor executive control, such as attention-deficit/hyperactivity disorder, obsessive–compulsive disorder, substance abuse, eating disorders, and pathological gambling (Chambers, Garavan, & Bellgrove, 2009; De Wit, 2009; Noël, Brevers, & Bechara, 2013; Robbins, Gillan, Smith, de Wit, & Ersche, 2012; Verbruggen & Logan, 2008b). In a recent article, Holmes, Craske, and Graybiel (2014) made a strong case for bridging the gap between basic laboratory research and clinical science and, more generally, for an integrative mental health science. They argued that evidence-based psychological treatments could benefit greatly from studying the mechanisms behind psychological treatments and from examining the processes that can relieve dysfunctional behavior. In the present study, we therefore probed the specific cognitive processes that regulate choice and high-level decision making. Ultimately, this could potentially open up new avenues for the treatment of pathological gambling.

Because one could argue that attributing the stop effect to some sort of cautiousness transfer is merely a redescription of the behavioral findings (Verbruggen, McLaren, et al., 2014), we report a series of experiments that examined how the introduction of stop signals influenced gambling. Experiment 1 tested whether stopping influenced decision making directly, by changing information-sampling styles, or indirectly, by changing arousal levels. Experiments 2a and 2b further explored the cautiousness transfer hypothesis by manipulating cautiousness in an unrelated secondary task. Experiment 3 examined whether the effect of stopping was a result of the requirement not to gamble on a proportion of the trials. In Experiments 4 and 5, we explored the generality of our findings. In Experiment 4, we asked whether the transfer effect was also observed in gamblers (with and without gambling problems); in addition, we performed two analyses that combined the data of all experiments using the bar task. Finally, in Experiment 5, we used a different gambling paradigm in which the exact probabilities of winning and losing were shown on each trial.

## Experiment 1: Is the Effect of Stopping on Monetary Decisions Driven by a Change in Processing Style or Arousal Levels?

In Experiment 1, we examined whether stop signals induced a more elaborate processing of stimuli and choice options in the gambling task. Previous work suggests that changes in processing styles occur when subjects expect a stop signal in a standard stop-signal task (Logan, Van Zandt, Verbruggen, & Wagenmakers, 2014; Verbruggen & Logan, 2009). This results in longer reaction times (RTs) but fewer choice errors. This has been attributed to an increase in response thresholds; consequently, more information has to be sampled before a decision is made (e.g., Ratcliff, 2006; Smith & Ratcliff, 2004). One possible mechanism by which the introduction of stop signals could have an effect on decision making in the gambling task is that the stop-signal manipulation causes subjects to process the various options more elaborately (i.e., more time focusing on the betting alternatives or actively considering more alternatives on each betting trial). In our task, we could not rely on RTs to estimate when a decision is made. The initial 3,500-ms phase in which the bars did not rise allowed for the possibility of subjects selecting an amount well before their choice response was executed. Therefore, we recorded eye movements as a measure of the temporal dynamics of decision making. We assumed that making a decision would correlate positively with dwell time, which is a measurement of how long people look at a specific region or amount on the screen. Changes in the overall dwell time without changes in its distribution over the options would suggest quantitative changes in processing style (as previously observed in, e.g., Verbruggen & Logan, 2009).

The introduction of stop signals could also change the sampling strategy in a more categorical or qualitative way. Recent work from our lab suggests that presenting stop signals alters processing of visual information in the primary go task (Verbruggen, Stevens, & Chambers, 2014). More specifically, we have demonstrated that in certain stop-signal tasks, proactive control also involves adjusting visuospatial attention parameters. The stimulus display of our gambling task required subjects to process visual information at various locations if they wanted to process all amounts to make a decision. Each bar turned black on stop-signal trials, so our stopping manipulation could have encouraged subjects to focus on each bar, and its associated amount, more systematically. In a risk-averse population,^2^ such a change could lead to a reduction in the amount people bet. Thus, systematic changes in sampling patterns (indexed by the distribution of average dwell time over locations or amounts and by the overall number of fixations) that correlate with changes in behavior would suggest such qualitative changes in sampling strategies.

Our first two accounts can be described as “cognitive” accounts that assume that stopping motor responses alters decision making by directly altering cognitive parameters. They are based on the idea that the executive control system regulates behavior by biasing or modulating the parameters of basic cognitive processes, such as stimulus processing and response selection (see the foregoing discussion). However, research on decision making under uncertainty, and gambling in particular, suggests that cognitive decision making and emotional processes may interact (Pessoa, 2013; but for a critical review of this area, see Dunn, Dalgleish, & Lawrence, 2006). Therefore, we also explored a third hypothesis in Experiment 1. Some studies have shown that stopping can change arousal levels (Casada & Roache, 2006; Jennings, van der Molen, Brock, & Somsen, 1992; van Boxtel, van der Molen, Jennings, & Brunia, 2001). By altering arousal levels, stopping may influence monetary decision making in our paradigm, much as Rockloff, Signal, and Dyer (2007; see also Rockloff & Greer, 2010) have shown that manipulating arousal can alter choice behavior in gambling tasks. We tested this “arousal” account by measuring skin conductance response (SCR), which provides a measure of autonomic arousal. If stopping influences gambling by altering arousal levels, SCR differences between load and no-load blocks should correlate with changes in betting strategies.

### Method

#### Subjects

Sixty-four volunteers (45 female, mean age = 21 years) from the University of Exeter (Exeter, United Kingdom) community participated for monetary compensation (£6 [approximately U.S.$9]), which was unrelated to performance. All experiments of the present study were conducted in accordance with the regulations laid out by the Exeter School of Psychology ethics committee, and written informed consent was obtained after the nature and possible consequences of the studies were explained. The target sample was decided in advance of data collection.

#### Apparatus, stimuli, and behavioral procedure

**Figure 1 fig1:**
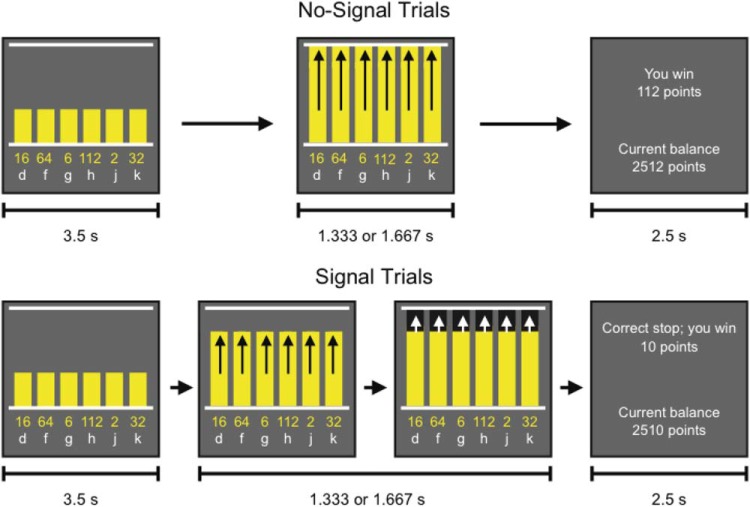
Examples of the two trial types in the bar task. The top panel shows the sequence of events on trials without signals. The bottom panel shows the sequence of events on a signal trial. The trial started with six potential bets. Underneath the betting options, letters were displayed that referred to the response keys on the keyboard. After 3,500 ms, the bars started rising until they reached the top white line after either 1.333 s or 1.667 s. On no-signal trials, subjects were required to choose one of the bets by pressing the corresponding letter when the bars reached the top white line. The response was recorded as correct when it was made between 250 ms before reaching the line and 250 ms after reaching the line. If the subjects won, they would get the points they bet; if they lost, they would lose half of the betted points. On signal trials, the bars turned black before reaching the line. If subjects saw the signal, then depending on the group they were in, they either made a double-response or attempted to withhold their response. On signal trials, subjects received or lost a fixed amount, depending on whether they responded correctly to the signal or correctly withheld their response. At the end of the trial, feedback was presented that showed the subjects how much they had won or lost and their current balance. On signal trials, subjects were told whether they responded correctly or correctly withheld their response, how much they gained or lost, and their current balance. See the online article for the color version of this figure.

The procedure was closely modeled on that of Verbruggen et al. (2013). Stimuli were presented on a 17-in. cathode ray tube monitor against a dark gray background (RGB: 100, 100, 100). The distance between the subjects’ eyes and the center of the screen was 58 cm. The task was run using Psychtoolbox (Brainard, 1997; Cornelissen, Peters, & Palmer, 2002). On each trial, six yellow (RGB: 255, 255, 0) vertical bars were presented next to each other (see Figure 1). Each bar was associated with a certain amount (presented in yellow) and a specific response key (presented in white: the *d*, *f*, *g*, *h*, *j*, or *k* key of a QWERTY keyboard). Subjects were instructed to select one of the amounts by pressing the corresponding key (e.g., in Figure 1, if they wanted to select 112, they had to press *h*). They were informed that the probability of winning decreased as the amount increased, without the exact probabilities being revealed. The amounts and response keys were presented below the bars. The order of the amounts varied from trial to trial to prevent spatial orienting toward one of the bars before the options were presented or response-bias effects (e.g., selecting higher amounts could reflect a rightward response bias if these were consistently presented on the right of the screen).

Each trial in *no-load blocks* started with the presentation of the start bars, amounts, and the associated keys (see Figure 1). The bars appeared between two white horizontal lines. After 3,500 ms, the bars started rising together. All bars reached the top line after 1,333 ms on low-bar trials and after 1,667 ms on high-bar trials (the distance between bottom and top line was approximately 9 cm on low-bar trials and 11 cm on high-bar trials). The original study manipulated bar height to test for effects of choice latency (see Verbruggen et al., 2012, supplementary material). Trials ended 500 ms after the bars reached the top line. Subjects had to execute the choice response before the end of the trial but not sooner than 250 ms before the bars reached the top line. The moving bars and response windows ensured that signals (see the following discussion) could be presented at an optimal moment. Feedback was presented at the end of each trial and indicated how much subjects had won or lost and what the current balance was. The feedback screen was then replaced by a blank screen after 2,500 ms, and the following trial started after a further 500 ms.

In *load blocks*, subjects had to select one of six amounts and indicate their choice when the yellow bars reached the top line on two-thirds of the trials, just as in the no-load blocks. On the remaining one-third of trials, the top of the rising bars turned black (*signal*) just before reaching the top line (see Figure 1). On signal trials, the subjects from the double-response group pressed the space bar of the keyboard with either thumb after they had indicated their choice (i.e., after they pressed the *d*, *f*, *g*, *h*, *j*, or *k* key of the keyboard). They had to press the space bar within 500 ms after the bars reached the top line. The subjects from the stop group had to refrain from making any response when the signal was presented. In both groups, signal onset was dynamically adjusted for each individual. Initially, the bars turned black 266 ms before they reached the top line. When subjects successfully stopped their response or pressed the alternate key in time (i.e., within 500 ms after the bars reached the top line), this delay was decreased by 33 ms, making it harder to successfully stop or execute the double-response on the next trial. When subjects failed to stop or execute the double-response in time, the delay was increased by 33 ms, making it easier to successfully stop or execute the double-response on the next trial.

On each trial in both block types, subjects could win or lose points. As noted earlier, subjects were informed at the beginning of the experiment that the probability of winning—p(win)—was lower for higher amounts, but we did not reveal the exact probabilities. The exact amount depended on the stake (low, medium, or high). The amounts [with p(win)s] subjects could win in the low-stake condition were as follows: 64 [p(win) = .20], 32 [p(win) = .25], 16 [p(win) = .325], 8 [p(win) = .47], 4 [p(win) = .605], and 2 [p(win) = .872]. In losses, subjects lost half the chosen amount. Amounts decreased exponentially to make the higher amounts more attractive. The expected values (EVs)—[EV = (p(win) × amount) − ((1 − p(win)) × amount/2)]—of the first three bets were positive and approximately the same. The two most “risky” options (Choice Options 5 and 6) had a negative expected value; we included these because superficially attractive options, associated with relatively high amounts but with a negative expected value, are common in gambling situations (for instance in the lottery, on racing odds, or slot machines). For medium stakes, all amounts were × 2; for high stakes, amounts were × 4. We manipulated stakes to increase selection demands, to encourage processing of the different amounts on each trial, and to encourage subjects to consider the relative risk versus benefit of each amount (Verbruggen et al., 2012). The three stakes occurred in random order with equal probability and had to be inferred by the subjects from the amounts that were presented below the bars. Because we could not infer which response subjects were planning to execute on successful stop-signal trials, the number of points won or lost on signal trials was fixed. Subjects won 10 points on successful signal trials and lost 10 points on unsuccessful signal trials in both the stop and double-response groups. Thus, on double-response response trials, subjects always won or lost 10 points, regardless of their choice response. Similarly, on unsuccessful stop trials, subjects always lost 10 points, regardless of the amount they indicated with their inappropriately executed choice response. On incorrect no-signal trials (i.e., trials on which no response was recorded, more than one response was recorded, or a key that was not part of the response set was pressed during the response window), subjects also lost 10 points.

The starting balance was 2,500 points. The experiment started with a short practice phase that consisted of a no-load block and a load block. The balance of points won or lost was reset after this practice phase. The experimental phase consisted of four no-load blocks and four load blocks of 36 trials each. Half of the subjects started with a load block, and the other half started with a no-load block. There was a short break after each block; block types alternated predictably, and their order was counterbalanced over subjects. Subjects were instructed to win as many points as possible. Unlike in our previous study, points were not converted to money at the end of the experiment. The aim in this experiment was to maximize the total number of points, and it is clear that the subjects tried to do so. Playing only for points is common in the literature (e.g., Knoch et al., 2006), which has shown it to be an effective incentive that helps to minimize the financial cost of the research. However, it is possible that removing the monetary incentive reduced the effect size (see the General Discussion).

#### Eye-tracking procedure

An EyeLink 1000 Desktop Mount camera system (SR Research, Ottawa, Ontario, Canada), calibrated before each block, tracked the gaze position of either the right or left eye during the whole block at a sampling rate of 500 Hz; each subject rested their chin in a chinrest for the duration of the testing. For most subjects, we tracked the right eye, but for 10 subjects we tracked the left eye because of difficulties in adequately capturing the right pupil or achieving satisfactory calibration.

#### SCR procedure

SCR was recorded using a Powerlab 26t setup with Biopac EL509 electrodes and LabChart 7 software (ADInstruments, Oxford, United Kingdom). Two electrodes were attached to the bottom side of the left wrist at the start of the experiment, before giving instructions and initializing the eye tracker configuration. This allowed ample time for the SCR signal to return to baseline.

### Analyses

#### Behavioral data

The primary dependent variable in the bar task is the betting score. The six available bets on each trial are ranked 1–6, with 1 being the lowest value. Higher betting scores indicated that subjects preferred higher amounts with a lower probability of winning. Averages were calculated for correct no-signal trials only: We excluded no-signal trials on which no response was recorded, more than one response was recorded, or a key that was not part of the response set was pressed during the response window. We excluded trials that followed an incorrect no-signal trial (see also Verbruggen et al., 2012), as they were infrequent and previous research suggests that such infrequent events could orient attention away from the main task (Notebaert et al., 2009).

In the original study, we tested whether the load effect increased or decreased during the experimental session (Verbruggen et al., 2012, supplementary analysis). Even though subjects were told that wins were less probable for higher amounts, the exact probabilities or expected values were not revealed. Our task therefore contained an element of learning. Further, proactive control often increases throughout the experiment (Verbruggen, Chambers, & Logan, 2013), which could modulate the load effect. To examine how the load effects evolved over time, we subdivided the session into four parts: Blocks 1 and 2 (first load- and no-load block, to be known as *Part 1*), Blocks 3 and 4 (second load- and no-load block, *Part 2*), Blocks 5 and 6 (third load- and no-load block, *Part 3*), and Blocks 7 and 8 (fourth load- and no-load block, *Part 4*).

We analyzed choice data using load (no-load vs. load blocks), stake (low, medium, high), and part (1–4) as within-subject variables and group (stop, double-response) as a between-subjects variable. We ran separate analyses of variance (ANOVAs) for the Group × Load × Stake interaction and the Group × Load × Part interaction because there were insufficient trials for a full factorial analysis.

In all experiments, we also calculated Bayes factors to explore the theoretically relevant effect of stopping. In Experiment 1, we calculated Bayes factors for the crucial Load × Group interaction. We also calculated Bayes factors for the simple main effect of stopping (Experiments 1, 2b, and 4) or the speed–accuracy and no-rise manipulations (Experiments 2a and 3). Both the interaction and the simple main effects can be tested using simple *t* tests (the first as a *t* test of difference scores, the second as a *t* test of performance in the no-load and load blocks). Several methods now exist to calculate the Bayesian equivalent of a *t* test. A Bayes factor compares two hypotheses; in this study, these are the hypothesis that introducing a stop load decreases betting scores (the experimental hypothesis) and the hypothesis that introducing a load does not influence betting (the null hypothesis). Bayes factors vary between 0 and infinity, with values of less than 0.33 indicating substantial support for the null hypothesis and values greater than 3 indicating substantial support for the alternative. Following Dienes (2011), in Experiment 1 of this study, we used a normal distribution with a mean of .15, which corresponds to the numerical difference in betting scores for the stop group in Experiment 1 of Verbruggen et al. (2012) and a standard deviation that is half of the mean. After Experiment 1, we adjusted the mean (.125)—and, consequently, the standard deviation (.0625)—by taking the average of the effect sizes observed in Verbruggen et al. (2012) and Experiment 1 of the present study. This acknowledges the fact that more information became available after the direct replication (i.e., Experiment 1 of this study). We calculated the Bayes factors using the R-version of Zoltan Dienes’ Bayes calculator (http://www.lifesci.sussex.ac.uk/home/Zoltan_Dienes/inference/bayes_factor.swf). All data files and R scripts used for the analyses are deposited on the Open Research Exeter data repository (http://hdl.handle.net/10871/15733).

#### Eye-movement data

**Figure 2 fig2:**
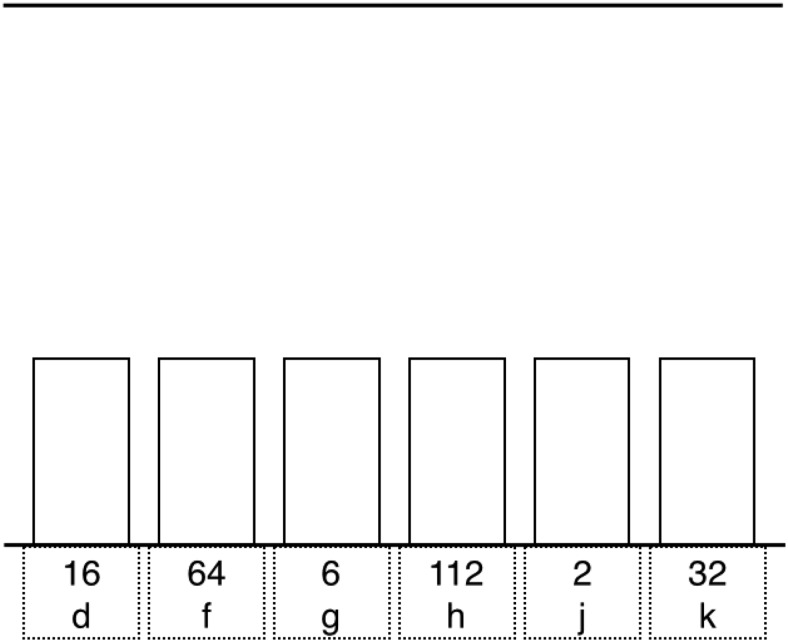
Regions (dotted lines) defined for the analysis of the acquired eye-tracking data made during the decision-making phase (0–3,500 ms). The size of each region was 90 × 99 pixels. For display purposes, we have only used black and white (see the Method section for a detailed description of the stimuli and their colors).

We selected six regions around the bets for analysis (see Figure 2 for their location and size). The eye-tracking data of two subjects were excluded because the tracker failed to properly track their pupil.[Fig-anchor fig2]

A first analysis focused on dwell time for each spatial location (from left to right, irrespective of amount). To assess whether scanning patterns changed in load blocks compared with no-load blocks, we measured the total duration of all the fixations on the six spatial regions in the decision-making phase (i.e., the 3,500-ms window between the presentation of the betting options and the onset of the bars rising). If subjects looked only at one region (e.g., if they fixated only the leftmost region) during the whole decision-making interval, the dwell time for this region would be 3,500 ms, and it would be 0 ms for all other regions. If they fixated other parts of the stimulus (e.g., the bars before they started rising), the sum of the dwell times would be less than 3,500 ms. The behavioral analyses (presented later) showed that the Group × Load interaction was not influenced by stake or part. Therefore, we collapsed across part and stake to increase the number of observations for each of the six regions. We compared dwell time for the six regions between groups and load conditions by running a three-way mixed ANOVA with spatial location, group (stop vs. double-response), and load (no-load vs. load block) as factors.

In a second analysis, we focused on dwell time for each amount (from low to high, irrespective of location). If subjects looked only at one amount (e.g., if they fixated only the number *8*) during the whole decision-making interval, the dwell time for this amount would be 3,500 ms, and it would be 0 ms for all other amounts. We compared dwell time for the six amounts between groups and load conditions by running a three-way mixed ANOVA with amount, group (stop vs. double-response), and load (no-load vs. load block) as factors. We again collapsed over stake and part to increase the number of observations.

Finally, we also report the average number of fixations on each a trial. The number of fixations was analyzed with a two-way mixed ANOVA with group (stop vs. double-response) and load (no-load vs. load block) as factors.

#### SCR data

To analyze possible effects on arousal, we compared SCR levels of load blocks with no-load blocks for both groups. SCR levels for each block were determined by averaging them over the 3,500-ms decision-making phase. By choosing this interval, we minimized the influence of movement artifacts that occur when a response is made. This analysis was done on correct no-signal trials (described earlier). To allow a direct comparison with the analysis of the eye-movement data, we focus on the Group × Load interaction only.^3^

### Results and Discussion

#### Manipulation checks

Approximately 49% of the signal trials across groups were correct, which confirmed the effectiveness of the tracking procedure (the target was 50%). There was a small but reliable difference (*p* = .035) in success rates on signal trials between the groups (failed double-responses = 48%, failed stops = 50%).

To test whether proactive inhibition induced motor cautiousness, we compared choice latencies in no-load and load blocks for both groups. RTs were calculated relative to the moment the bars reached the top line; consequently, negative values indicate that subjects responded before the bars reached the line. Subjects in the stop group were 73 ms slower in load blocks (63 ms) than in no-load blocks (−10 ms). This slowing was less pronounced in the double-response group (load blocks: 8 ms; no-load blocks: −35 ms; mean difference = 43 ms). This indicates that our stopping load induced motor cautiousness. There was a reliable effect of load (*p* < .001; see Table 1) and of group (*p* < .001) and a reliable Group × Load interaction (*p* < .001).[Table-anchor tbl1]

#### Betting data

A complete overview of the descriptive statistics and ANOVAs is given in Tables 2 and 3. Here, we focus on the theoretically relevant analyses. Subjects in the stop group selected lower bets with a higher probability of winning in load blocks (bet score = 3.13) than in no-load blocks (3.21). In contrast, subjects in the double-response group selected higher bets with a lower probability of winning in the load blocks (3.18) than in no-load blocks (3.08). The Group × Load interaction was reliable (*p* = .037, *B* = 5.57). This is consistent with the findings of Verbruggen et al. (2012) and demonstrates that the two load situations have a differential effect on choice: a stop load tends to decrease betting, whereas a double-response load tends to increase betting. Unlike in Verbruggen et al. (2012), the simple main effects of load failed to reach significance in both groups (stop: *p* = .22, *B* = 0.93; double-response: *p* = .08, *B* = 0.08).^4^ Betting scores tended to decrease over time, an effect that was more pronounced in the stop group. These conclusions are supported by a main effect of part (*p* < .001) and a Group × Part interaction (*p* < .001). However, part did not significantly modulate the Group × Load interaction (*p* = .14; see Table 3).[Table-anchor tbl2][Table-anchor tbl3]

#### Eye movements

**Figure 3 fig3:**
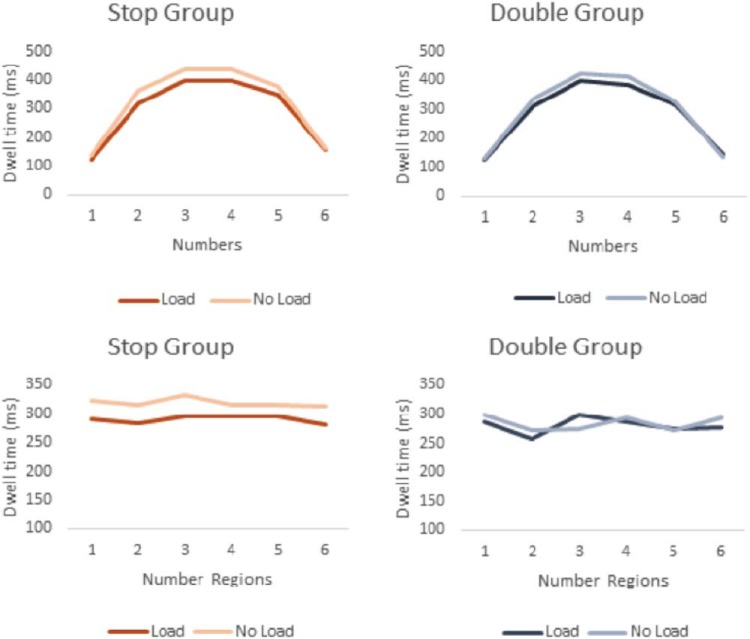
Dwell-time data from Experiment 1. The top panels show the mean dwell times during the decision-making phase for the six regions containing amounts arranged from left to right independently of the magnitude of the bet (1 being the leftmost region). In both groups, there was more dwell time for these regions in no-load blocks than in load blocks. There was also more dwell time for the more centrally presented bets. The bottom panels show the mean dwell times during the decision-making phase by amount irrespective of spatial location (1 being the lowest bet). See the online article for the color version of this figure.

As discussed in the introduction of this experiment, we hypothesized that if stop signals induced quantitative or qualitative changes in processing the amounts, dwell time in the load condition in the stop group should be higher per region/amount or more distributed over the six regions/amounts. The top panels in Figure 3 show the average dwell time for each location (from left to right, irrespective of amount), and the bottom panels show the average dwell time for each amount (from low to high, irrespective of location) as a function of group and load.[Fig-anchor fig3]

The analyses by spatial location (irrespective of amount) revealed an overall central-display bias: Subjects generally spent more time looking at the central areas than the periphery (*p* < .001; see Table 4). Further, subjects in both groups looked more at each number/response location in the no-load blocks than in the load blocks (*p* < .001). This likely reflects the differences in attentional monitoring demands in load blocks, in which subjects had to detect a signal that could appear close to the top line (see Verbruggen, Stevens, et al., 2014). The effect of load was larger for the central locations than for the noncentral locations (*p* = .018), although this could be attributable to a floor effect. It is important to note that Figure 3 shows that the dwell-time patterns were very similar for the stop and double-response groups. This conclusion is supported by the univariate analyses (see Table 4), which showed that the two-way Group × Load interaction (*p* = .17) and the three-way Group × Load × Location interaction (*p* = .97) were not significant. Thus, the location dwell-time data are inconsistent with the idea that stopping influenced gambling by encouraging a more elaborate processing style: Load generally decreased processing of amounts, and it did so independently of the kind of load (stop vs. double-response). Accordingly, it seems highly unlikely that the behavioral Load Type × Betting interaction was a result of differences in visual scanning.[Table-anchor tbl4]

The next analyses (by amount irrespective of spatial location) showed that subjects focused on each amount for approximately equal intervals of time (see Figure 3; main effect of amount: *p* = .084). This was true for both groups (*p* = .357) and both load conditions (*p* = .432). The Group × Load interaction (*p* = .171) and the three-way Group × Load × Amount interaction (*p* = .241) were nonsignificant. Combined, the dwell-time analyses indicate that the load manipulations did not induce an attentional bias toward lower or higher amounts.

Consistent with the dwell-time analyses, we found that the number of fixations was lower in load blocks (stop group *M* = 6.43, *SD* = 2.10; double-response group *M* = 6.30, *SD* = 2.17) than in no-load blocks (stop group *M* = 6.92, *SD* = 2.10; double-response group *M* = 6.34, *SD* = 2.28), *F*(1, 60) = 4.681, *p* = .034, η^2^ = .072). This difference tended to be more pronounced in the stop group, but the interaction was not reliable, *F*(1, 60) = 3.478, *p* = .067. There was also no main effect of group, *F*(1, 60) = .452, *p* = .504.

In summary, the eye-movement data are inconsistent with the increased cognitive processing accounts delineated in our introduction. The location and amount analyses indicated that load generally *decreased* dwell time on the relevant number regions. Furthermore, we did not detect any qualitative changes in scanning pattern. Combined, these findings indicate that a stop load did not induce a more elaborate or systematic processing style.

#### SRC analyses

SCR tended to be lower for the stop group (load *M* = .257 μS, *SD* = .16; no-load *M* = .261 μS, *SD* = .15) than for the double-response group (load *M* = .308 μS, *SD* = .11; no-load *M* = .300 μS, *SD* = .12). There was, however, no significant SCR difference between groups, *F*(1, 62) = .063, *p* = .82, generalized η^2^ = .001; no significant difference between no-load and load blocks, *F*(1, 62) = 1.831, *p* = .18, generalized η^2^ = .029; and no significant interaction between them, *F*(1, 62) = .643, *p* = .42, generalized η^2^ = .010. Thus, these findings are inconsistent with the idea that stopping influenced gambling by altering arousal levels.

## Experiments 2a and 2b: Can Transfer Effects Be Obtained Through Alternative Methods of Inducing Motor Caution?

The *motor cautiousness* hypothesis states that strategic control adjustments in the stop-signal task influence gambling, leading to a preference for lower amounts with a higher probability of winning. Motor caution can be manipulated in different ways. For example, many studies have shown that subjects respond more cautiously when they are instructed to respond as accurately as possible. We have previously argued that strategic adjustments in the stop-signal paradigm resemble such strategic speed–accuracy tradeoffs observed in other decision-making tasks. This raises the question whether effects of motor caution on gambling can be obtained in tasks that do not involve outright stopping of motor responses.

In Experiment 2a, we examined whether manipulating the speed–accuracy tradeoff modulated gambling. Subjects continuously alternated between the gambling task (without stop signals) and an unrelated perceptual decision-making task (also without stop signals). In the perceptual decision-making task, two gray rectangles were presented on each trial, and subjects had to respond to the location of the brighter rectangle. They could respond on all trials, but in half of the blocks (*speed* blocks), they were instructed to respond as quickly as possible to the gray squares, and in the remaining blocks, they had to respond as accurately as possible (*accuracy* blocks). Research on task switching has demonstrated that combining two tasks can produce strong carryover effects when people execute them on consecutive trials (Kiesel et al., 2010; Monsell, 2003; Vandierendonck, Liefooghe, Verbruggen, 2010). Usually, there are two costs associated with switching. First, performance is impaired when people switch from one task to another compared with repeating the same task (the task “switch cost”). Part of this cost can be attributed to inertia or interference caused by previously relevant task parameters or settings (Kiesel et al., 2010; Monsell, 2003; Vandierendonck et al., 2010). Second, performance on task-repeat trials is often worse in mixed blocks, in which both tasks occur, than in single-task blocks. At least part of this mixing cost is also attributable to competition between different possible rules (Vandierendonck et al., 2010). On the basis of the robust carryover effects observed in the task-switching literature, we predicted lower betting scores in accuracy blocks than in speed blocks: Control settings in the perceptual decision-making task were expected to influence choice in the gambling task, leading to more cautious betting (i.e., longer gambling latencies and a preference for lower amounts with a higher probability of winning) in accuracy blocks than in speed blocks.

To ensure that differences between the speed–accuracy manipulation and the stop manipulations in our previous experiments are not attributable to changes in the design, we also ran a task-switching experiment with the stop-signal task. In half of the blocks, subjects constantly alternated between the gambling task (without stop signals) and a stop-signal task; in the other half of the blocks, they alternated between the gambling task (without stop signals) and a choice RT task in which they could respond on all trials (without signals). If the effects of stopping in the previous experiments were a result of some inertia related to the stop rules or stop-specific control settings, subjects should prefer lower bets with higher probabilities of winning in stop-signal blocks than in no-signal (go) blocks.

### Method

#### Subjects

Sixty-four new volunteers (Experiment 2a: 32 subjects, 18 female, mean age = 20 years; Experiment 2b: 32 subjects, 22 female, mean age = 21 years) from the University of Exeter community participated for monetary compensation. In Experiment 2b, four subjects were replaced because their probability of responding on stop-signal trials was below .35, suggesting that the staircase tracking procedure (described later) did not work well for them. The exclusion criteria were decided in advance of data collection.

#### Procedure

In both experiments, subjects alternated between the gambling task and a secondary task. The gambling task was identical to the no-load blocks of Experiment 1.

##### Experiment 2a

In the secondary task (the perceptual decision-making task), two gray rectangles were presented, and subjects had to respond to the location of the brighter rectangle on all trials. The task always started with a task reminder (“Brightness”) for 250 ms, followed by the presentation of the two gray rectangles (width × height: 3.5 × 7 cm; distance between rectangles = 1.5 cm) in the center of the screen against a black background. One rectangle was darker than the other, and subjects responded to the location of the brighter rectangle by pressing the *s* (for left) or *l* (for right) key with the little finger of the left or right hand, respectively.

There were two block types: In the *accuracy* blocks, subjects were instructed to respond as accurately as possible to the gray rectangles, whereas they had to respond as quickly as possible to them in the *speed* blocks. We used staircase-tracking procedures in both block types to manipulate response strategies. In the accuracy blocks, the brightness level was continuously adjusted. After every four correct trials, the brightness difference (RGB difference) reduced by four RGB points (making the decision more difficult; e.g., RGB: 117, 117, 117 vs. RGB: 137, 137, 137 would become RGB: 119, 119, 119 vs. RGB: 135, 135, 135). The difference increased again after each incorrect trial (making the decision easier again). Feedback (presented for 1,000 ms) indicated to the subject whether the response was correct (“Brightness response = Correct”) or not (“Brightness response = Incorrect”). In the speed blocks, response latencies had to be shorter than a deadline that was continuously adjusted according to a four-down-one-up tracking procedure. The deadline decreased by 50 ms after four fast trials (making the speed task more difficult) but increased by 50 ms after one slow trial (making the speed task easier again). Feedback indicated whether the response was fast enough (“Brightness response = Fast enough”) or too slow (“Brightness response = Too slow”). The brightness difference (yoked to the difference in the accuracy blocks) remained constant in the speed blocks. The staircase procedures ensured that in both block types, the probability of positive feedback was approximately 84.1%.

The experiment started with two short blocks of 12 trials in which subjects could practice the perceptual decision-making task alone; the first block was always an accuracy block, followed by a speed block. This was followed by a short block of five trials in which subjects could practice the gambling task on its own. The main experiment consisted of 12 task-switching blocks of 24 trials in which the two tasks constantly alternated (i.e., there were no task repetitions). Half of the subjects started with a speed block, in which they switched predictably between the gambling task and the speed condition of the perceptual decision-making task. The other half started with an accuracy block, in which they switched predictably between the gambling task and the accuracy condition of the perceptual decision-making task. There was a short break after each block; block types were ordered in strict alternation. In the gambling task, subjects could (and were encouraged to) always respond. Subjects were informed at the beginning of each block whether they had to respond as quickly or accurately as possible in the perceptual decision-making task.

##### Experiment 2b

There were two conditions: no-signal (go) blocks and stop-signal blocks. In the *no-signal* blocks, no stop signals could occur in the secondary task. On each trial, two gray rectangles were presented in the secondary task. One rectangle was darker (RGB: 117, 117, 117) than the other (RGB: 137, 137, 137), and subjects responded to the location of the brighter rectangle by pressing the *s* (for left) or *l* (for right) key with the little finger of the left or right hand, respectively. The rectangles remained on the screen for 2,000 ms, regardless of RT. At the end of each trial, feedback was presented: “No Brightness response” when subjects failed to respond in time on no-signal trials, “Brightness response = Incorrect” when the response was incorrect, and “Brightness response = Correct” when the response was correct. Subjects could not win or lose points in the perceptual decision-making task. The feedback remained on the screen for 1,000 ms, after which it was removed. The next trial, which was always a gambling trial, started after 250 ms.

In *stop-signal* blocks, on one-third of the trials the gray squares turned blue (RGB: 0, 0, 255) after a variable delay, instructing the subjects to refrain from responding. The stop-signal delay was continuously adjusted according to a tracking procedure so that subjects would be able to stop on approximately 50% of trials (Logan et al., 1997; Verbruggen & Logan, 2009). When subjects made a response (signal-respond trial), the delay decreased by 50 ms on the following trial; when subjects successfully stopped (signal-inhibit trial), the delay increased by 50 ms on the following trial. Feedback was presented on no-signal trials (described earlier), signal-respond trials (“Try to stop your Brightness response”), and signal-inhibit trials (“Correct stop of Brightness response”).

The experimental procedure was identical to the procedure of Experiment 2a except that no-signal blocks replaced speed blocks and stop-signal blocks replaced accuracy blocks. Stop signals only occurred in the stop-signal task. In the gambling task, subjects could (and were encouraged to) always respond. Subjects were informed at the beginning of each block whether stop signals could occur in the perceptual decision-making task.

### Results and Discussion

#### Manipulation checks

In the perceptual decision-making task of Experiment 2a, subjects responded more quickly and made more errors in the speed blocks (mean RT = 493 ms, mean accuracy = .76) than in the accuracy blocks (mean RT = 691 ms, mean accuracy = .87). These differences were reliable; RT: *t*(31) = 8.76, *p* < .001, Cohen’s *d*_z_ = 1.54; accuracy: *t*(31) = 6.46, *p* < .001, Cohen’s *d*_z_ = 1.16. In the speed condition, 84% of responses were faster than the deadline, which demonstrates that the tracking was successful. Combined, these data show that subjects altered their speed–accuracy tradeoff, responding more cautiously in accuracy blocks than in speed blocks.

In Experiment 2b, subjects responded more slowly but more accurately to the gray squares in stop-signal blocks (mean RT = 688 ms, mean accuracy = 97.5%) than in no-signal blocks (mean RT = 565 ms, mean accuracy = 96.7%), which is consistent with our previous findings (Verbruggen & Logan, 2009). The RT difference was reliable, *t*(31) = 5.96, *p* < .007, Cohen’s *d*_z_ = 1.05, whereas the accuracy difference was not significant, *t*(31) = 1.28, *p* = .211, Cohen’s *d*_z_ = 0.23. Note that RT and accuracy differences between blocks were considerably smaller than the differences observed in Experiment 2a. On signal trials, the average probability of responding was .47, and the mean stop-signal delay was 413 ms.

#### Betting data

**Figure 4 fig4:**
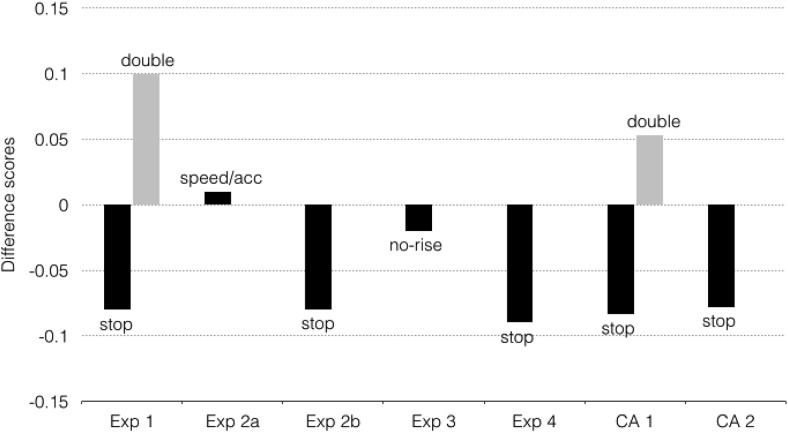
Differences between within-subject conditions (load vs. no-load, speed vs. accuracy [acc], rise vs. no-rise) in four experiments and in the combined analyses (CA). Exp. = experiment.

Overviews of the descriptive statistics and of the results of the mixed ANOVA are displayed in Tables 5 and 6 and Figure 4. In Experiment 2a, betting scores were very similar in accuracy blocks (betting score = 2.66) and speed blocks (betting score = 2.65; *p* = .81). Further Bayesian analyses showed that the data provide substantial support for the null hypothesis of no difference between accuracy and speed blocks (*B* = 0.14). There was a small but reliable difference in choice latencies: Latencies in the bar task were 7 ms longer in accuracy blocks (−22 ms) than in speed blocks (−29 ms), *t*(31) = 2.061, *p* = .048, Cohen’s *d*_z_ = .36. These results show that a block-based shift in speed–accuracy tradeoff does not influence gambling preferences. [Table-anchor tbl5][Table-anchor tbl6][Fig-anchor fig4]

In Experiment 2b, subjects preferred lower bets in stop-signal blocks (betting score = 2.82) than in no-signal blocks in which they could always respond (betting score = 2.9). This effect was significant (*p* = .050, *B* = 3.99). An analysis of choice latencies in the gambling task also showed a small but reliable carryover effect: Choice latencies were 9 ms longer in stop-signal blocks (29 ms) than in no-signal blocks (20 ms), *t*(31) = 2.85, *p* < .01, Cohen’s *d*_z_ = 0.50. These results show that switching between a neutral stop-signal task and a gambling task produces a transfer effect similar to introducing stop signals in the actual gambling game. This finding suggests that stopping can influence performance in other tasks, even when the tasks are separated in time. It is important to note that the absence of a difference between speed (viz., no-signal) and accuracy (viz., stop-signal) blocks in Experiment 2a indicates that outright stopping, over and above caution per se, is required to observe a transfer effect.

## Experiment 3: Is Response Inhibition Really Necessary for Inducing the Transfer Effect?

Experiments 2a and 2b indicate that a stop manipulation but not a speed–accuracy manipulation decreased betting. Experiment 3 further tested the specificity of the stop-signal manipulation. We examined whether the inclusion of trials on which subjects could not gamble was sufficient to produce an overall decrease in gambling. In Australia, Canada, and New Zealand, gambling-related pop-up messages on electronic gambling machines break play and inform gamblers when they have been playing continuously for a set period of time.^5^ Such messages may reduce gambling by encouraging players to actively decide to continue or discontinue their gambling session (Monaghan, 2008, 2009). Even when the message is noninformative, certain aspects of betting are influenced by the insertion of a break (Rockloff, Donaldson, & Browne, 2014). It is possible that introducing stop trials in a gambling task is similar to introducing a break, allowing subjects to actively decide to continue selecting higher bets with a lower probability or selecting lower amounts with a higher probability of winning instead.

To test the idea that inserting stop-signal trials acted as a break, we included blocks in which the bars did not rise on a third of the trials. On no-rise trials, subjects had to wait for the next trial (i.e., they could not place a bet). If the stop effect is a result of the inclusion of trials on which subjects could not place their bet, we should also see lower betting in no-rise blocks than in blocks in which the bars did rise on all trials.

### Method

#### Subjects

Thirty-two new subjects (22 female, mean age = 20 years) from the University of Exeter community participated for monetary compensation (£6 [approximately U.S.$9]), which was unrelated to performance.

#### Procedure

We used the bar task as described in Experiment 1. The only difference was that the load blocks were replaced by no-rise blocks. On one-third of the trials in the no-rise blocks, the bars would not start rising after 3,500 ms. The trial was automatically aborted when a normal trial would have ended. Subjects were instructed that they could and should only respond when the bars did rise and hit the top line. In this experiment, we compared betting scores of rise trials for standard blocks to the scores in no-rise blocks (analogous to no-signal trials in Experiment 1).

### Results and Discussion

An overview of descriptive statistics can be found in Table 2 and Figure 4. An overview of the ANOVAs is provided in Table 3. The betting scores were similar in standard blocks (betting score = 2.67), in which the bars did rise on all trials, and no-rise blocks (betting score = 2.65), in which the bars did not rise on a minority of the trials. A Bayesian analysis demonstrated that these data provided substantial support for the null hypothesis of no difference between the two block types (*B* = 0.30). These results show that when subjects do not gamble on a third of the trials, their choice behavior remains comparable to blocks in which they gamble on every trial. This suggests that the stop effect observed in Verbruggen et al. (2012) and in Experiment 1 is not driven by the insertion of trials on which subjects could not gamble. Instead, it highlights again that actively stopping motor responses is required to observe a transfer effect.

## Experiment 4: Does the Effect of Stopping on Decision Making Generalize to Different Populations?

In Verbruggen et al. (2012) and Experiments 1–3 of the present study, we used our bar task to examine the effects of a stop load on gambling in a university population. Before we can draw any theoretical conclusions, we believe it is important to demonstrate that the load effect is not population or task specific. In Experiment 4, we test whether we find the same load effect in gamblers using the bar task; in Experiment 5, we use a different gambling task to test whether the load effect can be found in other gambling tasks.

One of the implications of our results is that our stop-gambling task could be used to improve our understanding of gambling and possibly lead to a means of reducing problem gambling behavior. However, the university population tested so far is presumably risk averse and low in gambling experience (like most people in the population; Kahneman & Tversky, 1984). To examine whether the effect of stop signals is present in people who gamble more frequently, we ran an experiment in which we tested low-problem gamblers, high-problem gamblers, and matched controls using the same bar task as in Experiment 1.

### Method

#### Subjects

This study was conducted at Psychological Medicine Laboratory, CHU-Brugmann, Université Libre de Bruxelles (Brussels, Belgium). Forty-eight gamblers and 24 nongamblers participated in the study (see Table 7 for characteristics). Subjects from the nongambling control group were recruited by word of mouth from the community (e.g., hospital employees). To avoid biases resulting from inside knowledge of how these tasks operate, psychiatrists, psychologists, and other personnel with psychological training were excluded from participation. Gamblers were recruited through advertisements from the casino complex VIAGE in Brussels. The ads asked for subjects who “gambled frequently” to participate in a 1-day study to explore factors associated with gambling. A telephone screening interview was conducted by means of a locally developed screening tool (see also, e.g., Brevers, Cleeremans, Goudriaan, et al., 2012; Brevers, Cleeremans, Verbruggen, et al., 2012), which included an examination of frequency of gambling behavior and comorbid psychiatric disorders. We excluded any subject from the gambling groups who (a) reported gambling in casino settings less than once a week or less than four times a month during the past 18 months, (b) was older than 65 years (to avoid potential confounds from slow motor functioning due to aging), or (c) had experienced a substance abuse–related disorder during the year before enrollment in the study. In addition, those subjects included were judged to be healthy on the basis of their medical history. Substance use and medical history were examined using items taken from the Addiction Severity Index Short Form (Hendriks, Kaplan, van Limbeek, & Geerlings, 1989). We selected 24 frequent gamblers without a gambling problem and 24 gamblers with a gambling problem. Gambling dependence severity was assessed using the South Oaks Gambling Screen (SOGS; Lesieur & Blume, 1987). On the basis of Lesieur and Blume, a score of 5 or more on the SOGS was chosen as an indication of high-problem gambling. In other words, subjects in the *high-problem gambling* group scored 5 or more on the SOGS, whereas subjects in the *low-problem gambling* group scored 4 or less. Subjects in the matched control group reported that they did not gamble. The ethical review board of the Brugmann Hospital (Brussels) approved the study, and written informed consent was obtained from all subjects.[Table-anchor tbl7]

Six control subjects were replaced for various reasons (two were replaced because the percentage of anticipatory responses was higher than 15%; one was replaced because stop-signal delay was remarkably low—approximately 750 ms lower than the group average; and three were replaced because of technical issues). One high-problem gambler was replaced because the percentage of missed responses was higher than 15%. Inclusion of these subjects does not alter the results in a meaningful way.

#### Procedure

The procedure was the same as in the stop group in Experiment 1. We did not include a double-response group because of the potential vulnerability of this population to an increase in gambling behavior.

### Results and Discussion

#### Manipulation checks

As in Experiment 1, RTs were calculated relative to the moment the bars reached the top line; consequently, negative values indicate that subjects responded before the bars reached the line. Choice latencies were significantly longer in stop-load blocks (−11 ms) than in no-load blocks (−50 ms; *p* < .001). This confirms that our stopping load induced motor cautiousness. The difference between load and no-load blocks tended to increase over time (*p* < .001). Overall, high-problem gamblers (−9 ms) were slower than the control subjects (−39 ms) and low-problem gamblers (−43 ms). On signal trials, the average probability of responding was similar (control: 48%; low-problem gamblers: 47%; high-problem gamblers: 48%), which demonstrates that the tracking procedure was successful in all groups. We did not calculate stopping latencies because the procedure did not allow their reliable estimation.

#### Betting scores

An overview of the data and analyses is presented in Tables 2 and 3. On average, subjects preferred lower bets in load blocks in which signals could occur (load blocks; betting score = 3.52) than in no-load blocks (betting score = 3.61; *p* = .048, Cohen’s *d*_z_ = 0.23, *B* = 3.76). However, the Block Type × Part interaction suggests that this difference was present only in the later parts of this experiment (*p* = .035). The Load × Group interaction was not significant, but the three-way Group × Stake × Load interaction was (*p* = .029). This could indicate that the stop load did not influence gambling preferences in each group.

To further explore this three-way interaction, we calculated Bayes factors for each stake and group. In the control group, results were inconclusive for all three stakes (low stake: *B* = 2.11; medium stake: *B* = 1.24; high stake: *B* = 0.38). The combined Bayes factor was 0.99 (we multiplied the Bayes factors for each stake; note that we obtained a similar value when we collapsed stake first and calculated a Bayes factor based on these average scores). For the low-problem gamblers, results were inconclusive when the stakes were low (*B* = 1.68), but there was strong support for the experimental hypothesis when stakes were medium (*B* = 13.84) or high (*B* = 11.73). The combined Bayes factor in this case was 273.3. Thus, we can conclude that betting scores were lower in load blocks than in no-load blocks for low-problem gamblers, except when stakes were low. Finally, for the high-problem gamblers, we found support for the null hypothesis when stakes were medium (*B* = 0.16), but the results were inconclusive for low (*B* = 0.37) and high stakes (*B* = 2.01). The combined Bayes factor was 0.12, which suggests that stopping did not influence betting in this group (again, this was confirmed by a Bayesian analysis using the average betting scores). In summary, this experiment shows that occasionally stopping a response influenced betting strategies in low-problem gamblers but not in high-problem gamblers. The outcome for the control subjects was inconclusive.

There were other differences between groups. Betting scores were generally higher for high-problem gamblers (4.08) than for control subjects (3.43) and low-problem gamblers (3.18). Further, the *stake effect*, which refers to lower betting scores for higher stakes, was less pronounced in high-problem gamblers; this Stake × Group interaction was reliable (*p* = .007). This indicates that high-problem gamblers did not adjust their gambling strategies when the probability of losing larger amounts increased, unlike the two other groups. Finally, there was a significant Group × Part interaction. Betting scores decreased over time in the control group and the low-problem gambling group but not in the high-problem gambling group. The high-problem gamblers’ failure to adjust betting strategies when stakes increased and their failure to adjust betting strategies over time may be indicative of their gambling problems, thus reflecting less flexible decision-making processes in pathological gamblers (Brevers et al., 2012; Noël et al., 2013). It is important to note that the group differences also confirm the construct validity of our gambling task.

### Combined Analysis

In this experiment, the simple main effect of stopping was significant, but follow-up analyses indicated that this effect was only reliable in the low-problem gambling group. In addition, we have recently conducted an EEG study using the paradigm of Experiment 1. The results of this experiment are presented as supplementary material. There were 32 subjects per group (double-response vs. stop signal). In this study, we could not replicate the critical Group × Load interaction (no-load vs. load blocks), *F*(1, 62) = .43, *p* = .51. The main effects of group and load were also not significant (*p* > .86). Therefore, we did not analyze the EEG data.

The absence of an effect in the control group of this experiment and the failure to replicate the effect in another experiment raise the question of whether the effect of stopping on gambling is reliable. To examine this, we collapsed the data of all relevant experiments in two analyses (see Tables 8 and 9 and Figure 4) to test the Load × Group interaction (Analysis 1) and further explore the simple main effect of stopping (Analysis 2).[Table-anchor tbl8][Table-anchor tbl9]

For the first analysis, we collapsed the data of (a) Experiment 1 of Verbruggen et al. (2012), (b) the replication study discussed in Verbruggen et al. (2012), (c) Experiment 1 of the present study, and (d) the EEG experiment. In these experiments, there were both stop and double-response groups. This resulted in a sample size of 216 unique subjects (108 in each group). To examine the time course, we compared the first half with the second half of the experiment, because the number of blocks differed between experiments.^6^ The betting scores were analyzed using a Group (stop vs. double-response) × Load (no-load vs. load) × Stake (Low, medium, or high) × Part (first half vs. second half) mixed ANOVA. For an overview of the descriptive and inferential statistics, see Tables 8 and 9 and Figure 4. The combined analysis shows that subjects in the double-response group tended to go for higher amounts in load blocks (betting score = 2.95) than in no-load blocks (betting score = 2.89), whereas subjects in the stop group chose lower bets in load blocks (betting score = 2.81) than in no-load blocks (betting score 2.89). This critical interaction is reliable (*p* < .01), but the effect size is small (see Table 9; Cohen’s *d* based on a *t* test for the interaction = 0.35). Note that the mean betting scores of the two groups were the same in no-load blocks. The simple main effect of load was significant in the stop group, *F*(1, 107) = 4.33, *p* = .0397, generalized η^2^ = .001, but not in the double-response group, *F*(1, 107) = 2.72, *p* = .10, generalized η^2^ < .001.

In the second analysis, we further explored the simple main effect of stopping on gambling. We collapsed the data of the stop groups of (a) Experiment 1 of Verbruggen et al. (2012), (b) the replication study discussed in Verbruggen et al. (2012, p. 814), (c) Experiment 1 of the present study, (d) the EEG experiment, and the data of (e) Experiment 2b and (f) Experiment 4 of the present study. This resulted in a sample of 212 stop subjects. The betting scores were analyzed using a Load (no-load vs. load) × Stake (low, medium, or high) × Part (first half vs. second half) repeated measures ANOVA. Overviews of the descriptive and inferential statistics are contained in Table 8 and Figure 4. Overall, subjects selected lower bets in blocks in which a stop signal could occur (betting score = 3.10) than in blocks in which they could always respond (betting score = 3.17; *p* = .008), but the effect size was small (see Table 9; Cohen’s *d*_z_ calculated on the basis of a paired *t* test = 0.21). This difference between block types tended to be more pronounced in the second half of the experiment (difference = .12) than in the first half (difference = .04; *p* = .039). In a follow-up analysis, we examined whether betting was influenced by the signal properties of the previous choice trial (stop-signal vs. no-signal; we excluded the data of Experiment 2b from this analysis because the signals were not present in the actual gambling task in this experiment). People often slow down after a stop trial (e.g., Bissett & Logan, 2011; Rieger & Gauggel, 1999; Verbruggen & Logan, 2008b). Such sequential effects suggest that response strategies and control settings set at the beginning of a block are further adjusted after a signal trial. However, sequential effects of stopping did not significantly modulate choice behavior in load blocks: Betting scores after a signal trial (3.0977) were very similar to betting scores after a no-signal trial (3.0979), *F*(1, 179) < .01, *p* = .99, generalized η^2^ < .0001.

**Figure 5 fig5:**
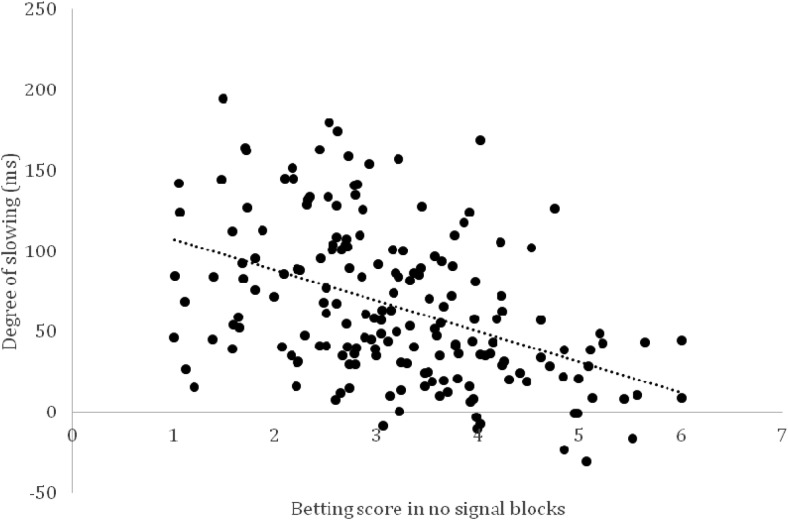
There was a negative correlation (*r* = −.44) between choice behavior in no-load blocks and the amount of slowing in stop-signal blocks.

Finally, we explored correlations between the size of the stop effect (the score of load blocks minus the score of no-load blocks; negative values indicate that people selected lower bets in load blocks than in no-load blocks), baseline risk taking in no-load blocks as indexed by betting score, and the degree of slowing in the bar task (reduction in RT in stop blocks compared with go blocks). Experiment 2b of this study was also excluded from this analysis, resulting in a sample of 180 subjects. The size of the stop effect did not correlate significantly with slowing (*r* = .05, *p* = .49) or the betting score in no-load blocks (*r* = .01, *p* = .90). However, there was a significant correlation between the betting score in no-load blocks and the degree of slowing (*r* = −.44, *p* < .001; see Figure 5). This is consistent with our recent finding that stop-signal latencies in a standard stop task correlate with risk taking in no-load blocks of the bar task (Verbruggen et al., 2013), and it provides further support for the idea that there is some overlap between motor control and risk taking in our gambling task.[Fig-anchor fig5]

## Experiment 5: Does the Effect of Stopping on Decision Making Generalize to Other Tasks?

In Experiment 4, we explored whether the stop-load effect in the bar task generalized to different populations. The bar task measures decision making under uncertainty (i.e., the exact probabilities of winning were unknown). In this final experiment, we combined the stop-signal manipulation with a task that measures decision making under risk (Porcelli & Delgado, 2009). On each trial, subjects chose between two options of equal expected value framed in terms of “wins” or “losses.” In the win domain, subjects could win points (e.g., 80% chance of winning £0.75 vs. 20% chance of winning £3.00), whereas they could lose points in the loss domain (e.g., 80% chance of losing $0.75 vs. 20% chance of losing $3.00). People generally tend to take more risks when decisions are framed in terms of losses (Kahneman, 2003). Therefore, in this experiment, we could explore whether stopping influences decision making equally in the win and loss domains.

### Method

#### Subjects

Thirty-six volunteers (29 female, mean age = 19 years) from the University of Exeter community participated for partial course credit or monetary compensation (£5 [approximately U.S.$7.5]), which was unrelated to performance. For every 10 subjects, an extra £5 was given to the subject with the highest end score. One subject was excluded because the number of missed responses was too high (34%), and three other subjects were excluded because p(respond | signal) was either higher than 75% or lower than 25% (indicating that the tracking procedure did not work properly). Thus, 32 subjects were included in the final analysis.

#### Apparatus, stimuli, and behavioral procedure

Stimuli were presented on a 17-in. liquid crystal display monitor against a white background. The task was run using Psychtoolbox (Brainard, 1997). Subjects were tested in large groups, so we included extra training blocks with immediate feedback to ensure that all subjects understood the tasks.

**Figure 6 fig6:**
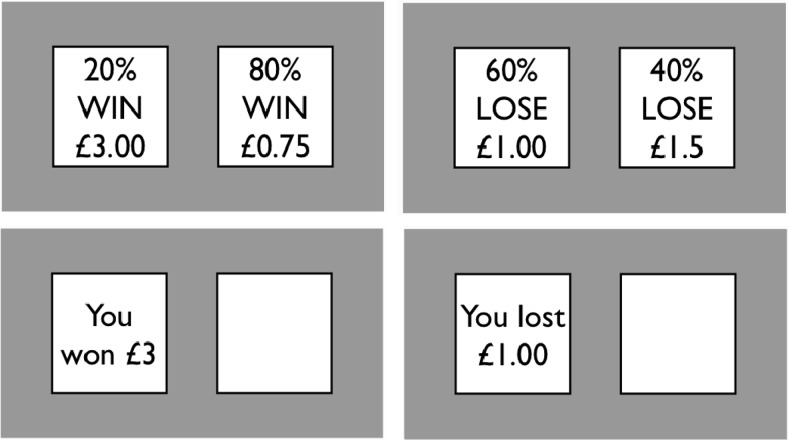
Examples of cards from the risk task.

In the first training phase of the experiment, subjects undertook the risk task. On each trial, a subject saw a pair of cards labeled either “WIN” or “LOSE” (see Figure 6, top panels). Each card mentioned an amount of money and a probability (in percentage). On WIN trials, the cards represented the amount that the subject could win and the probability of winning that amount. On LOSE trials, the cards represented the amount the subject could lose and the probability of losing that amount. On both WIN and LOSE trials, the higher amounts of money were associated with lower probabilities of winning or losing, respectively (we henceforth refer to the these cards as the “risky options”). There were four possible card combinations: *20% chance to win £3* versus 80% chance to win £0.75, *40% chance to win £1.50* versus 60% chance to win £1.00, *20% chance to lose £3* versus 80% chance to lose £0.75, and *40% chance to lose £1.50* versus 60% chance to lose £1.00 (the risky options are italicized here for expository purposes). On each trial, the expected value of the two options was the same. The cards were displayed for up to 2,500 ms, during which the subjects could select one of the two cards by pressing the left arrow button for the left card or the right arrow button for the right card. After a choice had been made, the computer immediately showed the subject the “other side” of the chosen card, on which the result of the bet was displayed for 1,000 ms (see Figure 6, bottom panels). The next trial started after 500 ms. This training phase consisted of three blocks of 16 trials. Each card combination occurred four times per block, and the order of presentation was randomized.[Fig-anchor fig6]

In the second training phase, subjects performed a neutral stop-signal task. In no-signal blocks, on each trial the computer displayed two cards next to each other (analogous to the risk task), one with a *−* on it and another with a *+* on it. Subjects were instructed to respond as quickly as possible to the location of the + card by pressing the corresponding key. The cards remained on the screen until a response was executed or 2,500 ms had elapsed. At the end of each trial, feedback was presented for 1,000 ms: “Too late!” when subjects failed to respond in time on no-signal trials, “‘Wrong Key!” when the response was incorrect, and “Correct” when the response was correct. The next trial started after 500 ms. In signal blocks, stop signals (a short auditory tone) occurred on 25% of the trials, instructing the subjects to withhold their response. The auditory signal was presented after a variable delay, which was continuously adjusted in 50-ms steps according to a tracking procedure to obtain a probability of stopping of .50 (see Experiment 1). On signal trials, the stimulus remained on the screen until a response was executed or until stop-signal delay + 1,250 ms had elapsed. This second training phase consisted of eight blocks (four no-signal [NS] blocks and four signal [S] blocks) of 16 trials. The order of the blocks (NS-S-NS-S-NS-S-NS-S or S-NS-S-NS-S-NS-S-NS) was counterbalanced, and subjects were informed at the beginning of each block whether stop signals could occur.

In the third phase (the test phase), subjects played the same risk task as in Phase 1, but half of the blocks were signal blocks in which auditory stop signals occurred on 25% of the trials (cf. Phase 2). This test phase consisted of six blocks (three no-signal blocks and three signal blocks) of 16 trials. Again, the order of the blocks was counterbalanced, and subjects were informed at the beginning of each block whether stop signals could occur.

### Results and Discussion

#### Manipulation check

Analyses of the stop-signal training phase (Phase 2) show that subjects responded more accurately (.01 difference; *p* < .01) but more slowly (121-ms difference) in signal blocks than in no-signal blocks (*p* < .001). This indicates that subjects responded more cautiously in signal blocks than in no-signal blocks in the training phase.

#### Risk scores

To examine performance in the test phase (Phase 3), we calculated the proportion of trials on which subjects selected the risky option (the option with the higher absolute amount but lower probability of winning or losing; described earlier). In the loss domain, subjects tended to prefer the risky option more in the signal blocks (*M* = .52, *SD* = .23) than in no-signal blocks (*M* = .51, *SD* = .24). In contrast, in the win domain, subjects preferred the safe option more in signal blocks (*M* = .43, *SD* = .26) than in no-signal blocks (*M* = .47, *SD* = .24). There was no reliable effect of load (*p* = .280) or domain (*p* = .327). The interaction also failed to reach significance (*p* = .11; see Table 10). Nevertheless, we ran *t* tests and calculated Bayes factors to test whether there were reliable simple main effects of signal-block type in each domain. To calculate the Bayes factor, we used a prior distribution of possible effect sizes with the mean of 0.03 and standard deviation of 0.015 because the combined analysis suggested that stopping could decrease risk taking by approximately 3%. The effect of stop signals (the signal vs. no-signal block difference) in the win domain was reliable, *t*(31) = 2.09, *p* = .045, Cohen’s *d*_z_ = .37, *B* = 5.95. In contrast, this difference in the loss domain was not reliable and supported the null hypothesis, though not unequivocally, *t*(31) = −0.466, *p* = .645, Cohen’s *d*_z_ = .08, *B* = 0.37. Note that Bayes factors below 1/3 provide substantial support for the null hypothesis. Bayes factors between 1/3 and 1 provide anecdotal evidence for the null hypothesis. In sum, the results of this experiment tentatively suggest that the inclusion of stop signals in a risk task leads to reduced risk taking but only in the win domain; in the loss domain, the inclusion of the stop signals does not seem to affect choice behavior much. We discuss the implications of these findings in the General Discussion.[Table-anchor tbl10]

## General Discussion

Recently, we reported that adding a cognitive load to a gambling task influences monetary decision making (Verbruggen et al., 2012). When we asked people to occasionally add a response in a secondary task, they tended to prefer higher bets with a lower probability of winning. This is consistent with previous studies demonstrating that a cognitive load increases impulsivity or random responding in delayed discounting tasks (Franco-Watkins, Pashler, & Rickard, 2006; Franco-Watkins, Rickard, & Pashler, 2010; Hinson, Jameson, & Whitney, 2003). In contrast, when we asked people to occasionally withhold a motor response in the secondary task, they tended to prefer lower bets with a higher probability of winning (Verbruggen et al., 2012). In this article, we further explored the link between motor control and gambling.

### Effects of Stopping Motor Responses on Gambling

The main aim of this study was to examine how stopping motor responses could influence gambling-related decisions. Dealing with stop signals is thought to require reactive and proactive inhibitory control (Aron, 2011; Logan, 1994; Verbruggen & Logan, 2009). Subjects need to engage in reactive inhibitory processes when a stop signal occurs. When subjects are informed that they may have to stop in the near future, they are thought to engage in proactive control. In Experiment 1, we examined whether proactive control adjustments influenced gambling by changing information-sampling strategies. One possibility is that the presence of stop signals leads to more comprehensive processing of the betting options (cf. Verbruggen & Logan, 2009). Further, recent work in our lab has demonstrated that adding visual stop signals to a task alters visuospatial attentional settings (Verbruggen, Stevens, et al., 2014). Such changes could influence betting, as our task required processing amounts presented at different locations on the screen. We also tested a noncognitive account in this experiment. Stop signals have been shown to alter arousal levels (Casada & Roache, 2006; Jennings et al., 1992; Van Boxtel et al., 2001), which could influence gambling (Rockloff et al., 2007; Rockloff & Greer, 2010). The results of Experiment 1 were inconsistent with the processing and arousal accounts. The patterns of eye fixations and SCRs were similar in the stop and double-response groups, which is contrary to what one would expect if stop signals induced a more elaborate processing style.

Experiment 2a did not support a general motor caution account either. We found that a speed–accuracy tradeoff in a secondary choice-reaction task without stop signals did not modulate gambling preferences (even though gambling latencies were influenced). The absence of a transfer effect could not be attributed to the use of a task-switching design, because switching between a secondary stop-signal task and the gambling task (without stop signals) did modulate gambling (Experiment 2b). In Experiment 3, we showed that occasionally not being able to make a bet (instead of encountering a stop signal) did not influence decision making either. Previous work has demonstrated that inserting a break between gambles can reduce betting by encouraging people to actively consider whether to continue gambling or not. However, introducing trials on which subjects could not gamble did not change betting in the bar task. The results of Experiments 2 and 3 provide strong support for the idea that the presence of stop signals is essential for the transfer.

In Experiment 4, we tested whether the effect was also found in low-problem gamblers, high-problem gamblers (i.e., people for whom gambling was a problematic habit), and matched controls. The high-problem gamblers took more risks in the bar task than control subjects (who did not gamble), which confirms the construct validity of our paradigm. The low-problem gamblers showed a reliable reduction in betting scores in blocks in which stop signals could occur (load blocks) versus blocks in which they could always respond (no-load blocks). This demonstrates that the effect of stopping generalizes to the wider, nonstudent population. However, the high-problem gamblers did not show a similar effect. Of course, this could be a by-product of, or a causal factor in, their gambling problem. In addition to the analyses of the three groups, we ran two analyses combining all the behavioral bar-task data we have collected in the past 3 years. These analyses established that cognitive load influences gambling, although the effect size was small. Further, we found a correlation between motor cautiousness and gambling in no-signal blocks, which provides further support for the link between gambling and motor control (see also Verbruggen, Adams, et al., 2013).

Finally, Experiment 5 suggested that the carryover effect is not unique to the bar task. We observed reduced risk taking when stop signals could occur in the win domain but not in the loss domain. However, this difference between domains should be interpreted with some caution as the interaction failed to reach significance. Further, Bayesian analyses provided strong support for the experimental hypothesis in the win domain (*B* was larger than 3) but weaker evidence for the null hypothesis in the loss domain (*B* was slightly larger than 1/3). In the supplementary material, we report the results of another experiment in which we introduced stop signals in a slot-machine gambling task. The results of this experiment were generally consistent with the results found in the bar and risk tasks. Together, these two experiments indicate that the stop-load effect can be observed in different gambling tasks, but the effect size remains small.

### A Search for Common Mechanisms

Our results indicate that stopping influences gambling, but we have found little evidence for changes in decision-making strategies. How does stopping influence choice? On the basis of our recent review of the literature on learning and response inhibition (Verbruggen, Best, Bowditch, Stevens, & McLaren, 2014), we propose that response inhibition reduces the hedonic and motivational value of stimuli. Work by Guitart-Masip, Talmi, and Dolan (2010) suggests that there may be a hardwired link between reward (or approach) and going and between punishment (or avoidance) and stopping. A similar link was suggested by Spunt, Lieberman, Cohen, and Eisenberger (2012), who showed that stopping causes affective distress. Further, several studies have found that consistent pairing of stimuli to stopping in a go/no-go or stop-signal-paradigm reduces subsequent consumption or approach behavior toward them (Houben, 2011; Houben, Havermans, Nederkoorn, & Jansen, 2012; Houben & Jansen, 2011; Jones & Field, 2013; Lawrence, Verbruggen, Morrison, Adams, & Chambers, 2014; Veling, Aarts, & Papies, 2011; Veling, Aarts, & Stroebe, 2013). Jones et al. (2011) showed that these effects are not related to changes in heart rate, blood pressure, or self-reported changes in general mood. Instead, the reduction of consumption or approach behavior may be attributable to devaluation of the stop or no-go stimuli (Houben et al., 2012; Kiss, Raymond, Westoby, Nobre, & Eimer, 2008; Veling, Holland, & van Knippenberg, 2008) and to reductions in motivational value (Ferrey, Frischen, & Fenske, 2012). Response inhibition may affect the motivational value of stimuli via the creation of links between the stimuli and the appetitive/aversive centers postulated by Dickinson and Dearing (1979). These two centers mutually inhibit each other, which could account for a wide range of phenomena in the learning literature (cf. Dickinson & Balleine, 2002). For example, Dickinson and Lovibond (1982; as cited in Dickinson & Balleine, 2002) demonstrated that a conditioned appetitive jaw movement could be suppressed by an aversive defensive eyeblink in rabbits; this interference was attributed to an inhibitory interaction between an appetitive center and an aversive center. The link between stopping and aversion could easily explain why the value of stimuli associated with stopping, or consumption of the stop-related items, decreases. Further, priming of the aversive or avoidance center could also explain why being cautious in a stop-signal task with neutral stimuli reduces subsequent alcohol consumption in a taste test (Jones et al., 2011).

Having to stop responses regularly in the context of a gambling task could generally reduce gambling via a similar mechanism: By activating or priming the aversive center, approach behavior toward the higher amounts is reduced, and subjects develop a preference for the safer options. The appetitive/aversive centers account can explain why stopping influences gambling in the bar task (Experiments 1,^7^ 2b, and 4), whereas the speed–accuracy manipulation did not (Experiment 2a). The no-rise manipulation in Experiment 3 (which was possibly the equivalent of a no-go manipulation) did not produce an effect on gambling either, but this could be due to no-rise trials being less aversive than stop-signal trials, which required last-minute stopping of a prepared response. The behavioral trends observed in Experiment 5 are also consistent with the appetitive/aversive account: Stopping reduced risk taking when subjects had to choose between two options framed as wins (appetitive), but not when they were framed as losses (aversive). In fact, there was a slight numerical increase in risk taking in the loss domain. We propose that stopping primes or activates the aversive center, which suppresses the appetitive center. Thus, when choice options are framed as wins, the activation of the aversive center will reduce approach toward the high (appetitive) amounts; in contrast, in the loss domain, stopping-induced priming of the aversive center could make losses even more aversive, leading to increased risk taking to avoid them (though the difference between load and no-load blocks was not reliable in the loss domain). Finally, the appetitive/aversive centers account may provide further insights into the data for high-problem gamblers. The absence of a load effect for the low and medium stakes in high-problem gamblers could reflect an imbalance between the two centers. The results indicate that high-problem gamblers approach the high amounts more often (as indicated by the high betting scores). Further, previous research indicates that at least some subpopulations of high-problem gamblers fail to properly activate the stopping network (e.g., Brevers et al., 2012). The appetitive and aversive centers mutually inhibit each other, so increased activation of the appetitive center and a failure to properly activate the stopping network on stop trials would lead to the absence of a stop-load effect.

The idea that aversive stimuli or events can reduce gambling also receives support from other studies. For example, Guitart-Masip, Talmi, and Dolan (2010) have demonstrated that cues associated with negative events in a learning phase can reduce subsequent gambling in a test phase. Similarly, Clark et al. (2012) have shown that cues associated with the delivery of a shock (i.e., an aversive event) reduce risky decision making in a gambling task. Future research is required to explore the “stopping is aversive” conjecture and the extent to which this can account for the transfer between the stop and gambling tasks. But at this point, it seems to provide a parsimonious account for a wide range of findings related to stopping and conditioned inhibition.

### Limitations

Together, our studies show that stopping motor responses and gambling interact. However, the observed effect size was small. The small size of the effect complicates further investigation of the underlying mechanisms and makes possible applications in the clinical domain more questionable. Therefore, further investigations should aim to increase the effect size.

One potential reason for the small effect size is that subjects played for points rather than a monetary reward (although in one pilot study we found that large monetary rewards actually decreased overall gambling in students, as they all selected the safe amounts that in their opinion were still substantial compared with the average pay in a psychology experiment). In Experiment 5, the binary nature of the dependent variable could have further reduced statistical power. Second, the large individual differences in the way subjects play gambling games could have influenced the effect size. Different strategies may lead to a differential reward history, which might in turn influence later gambles. Only when gambling games are played for a sufficiently long time might the reinforcement schedules become more similar. Third, the size of the effect could be influenced by the populations we used in our studies. Most people tend to be risk averse under uncertain conditions. Except for Experiment 4, all of our experiments were run with university students; if our population does not gamble often and already takes very little risk, the effect of a manipulation that aims to further reduce risk taking is already somewhat constrained. Studies using stop-signal training to reduce food consumption show that the effect of stop training particularly affects people with less inhibitory control in comparison with people with higher levels of inhibitory control (Houben, 2011; Veling et al., 2011). Consistent with this finding, we found that the size of the effect was numerically larger for the low-problem gamblers than for the matched controls, though this group difference was not reliable. High-problem gamblers, however, did not show a stop effect, possibly for reasons discussed earlier.

### Practical Implications

Despite the limitations, we believe that our findings and the theoretical framework presented here can have some practical implications. Our work indicates that stopping motor responses can encourage people to select lower bets with a higher probability of winning. We attribute this to a reduction in approach behavior via the priming of an aversive or avoidance center. This could open avenues for the development of a behavioral treatment program for problem gambling. For example, interventions could consistently pair gambling tasks or gambling-related stimuli with stopping. Previous work has already shown that similar response-inhibition training tasks can influence food and alcohol consumption. For example, Veling, van Koningsbruggen, Aarts, and Stoebe (2014) found that an online training program that involved practicing a food-related response-inhibition task for 4 weeks was an effective tool to induce weight loss. In other words, this finding demonstrates that training response inhibition may have an impact on behavior outside the lab.

Training response inhibition may in fact be useful for the treatment of various clinical populations. Response inhibition deficits have been observed in impulsivity disorders, such as attention deficit/hyperactivity disorder, and in compulsivity disorders, such as obsessive–compulsive disorder (Robbins et al., 2012). Inhibition deficits have also been observed in substance abuse, eating disorders, and gambling disorders (note that impulsivity and compulsivity may both be present in addictive disorders; Robbins et al., 2012). The present study and work on “stop learning” indicates that behavioral training tasks may be used to alleviate such response-inhibition deficits. Of course, much more work is required to understand how inhibition training works, to determine the longevity of the training effects, and to know for which disorders or subpopulations inhibition training tasks can be useful. For example, response-inhibition training may not be beneficial for all pathological gamblers. Blaszczynski and Nower (2002) have argued that there are several pathways to problem gambling. They distinguished between three subgroups of pathological gamblers: (a) behaviorally conditioned problem gamblers; (b) emotionally vulnerable problem gamblers; and (c) antisocial, impulsive problem gamblers. They proposed that different treatments may be required for the three subgroups. Indeed, stop-signal training may be more effective for behaviorally conditioned or impulsive than for emotionally vulnerable problem gamblers. More generally, this highlights again the need for a detailed analysis of control deficits in clinical populations (Holmes et al., 2014; Verbruggen, McLaren, et al., 2014).

Of course, stop-signal training will not be a “silver bullet” for the treatment of any impulsivity and compulsivity disorders. However, it may complement other existing treatments (see, e.g., Veling et al., 2014, who used a combined approach in their food-training study), including those for problem gambling. For example, therapies targeting erroneous cognitions, gambling urges, and motivations appear efficacious in the treatment of problem gambling (Potenza et al., 2013, p. 381). Stop-signal training alters habitual responses or behaviors and can change the motivational value of stimuli, tasks, or contexts. As such, it may become an extra clinical tool for the treatment of problem gambling. It may also complement regulatory measures. As noted earlier, in certain countries, gambling-related pop-up messages on electronic gaming machines interrupt play. The gambling-related pop-up messages may help to remind people about their gambling-related goals (e.g., “I should not spend more than an X amount of money”) and encourage them to actively decide whether they would like to continue gambling or not, whereas our stop-signal manipulation seems to reduce betting and approach behavior via a link with an aversive system. In other words, the pop-up messages and the stop-signal training may provide two different routes to modulate decision making when gambling and could, therefore, complement each other well.

### Conclusions

The results of several experiments support a link between motor control and decision making. They indicate that the effect of response inhibition on gambling is present in different populations and tasks and that it is driven by the presence of stop signals specifically. Despite the modest effect size, this link between stopping and gambling provides a strong incentive to explore other avenues in an attempt to increase our understanding of risky decision making in humans. It also perhaps suggests interventions to reduce harmful behavior.

## Supplementary Material

10.1037/xap0000039.supp

## Figures and Tables

**Table 1 tbl1:** Results of the Analysis of Variance of the Choice Latencies in Experiments 1 and 4

Experiments and factors	*df*_1_	*df*_2_	*F*	*p*	Gen. η^2^
Experiment 1					
Group	1	62	9.375	.003	.116
Load	1	62	127.920	.000	.219
Group × Load	1	62	8.985	.004	.019
Experiment 4					
Group	2	69	3.368	.040	.080
Load	1	69	87.273	.000	.125
Group × Load	2	69	0.932	.399	.003
*Note.* *df* = degrees of freedom; Gen. = generalized.					

**Table 2 tbl2:** Overview of the Betting Scores (Means, With Standard Deviations in Parentheses) as a Function of Stake and Part for Experiments 1, 3, and 4

Experiments and conditions	Stake			
	Low	Medium		High
Experiment 1				
Stop load	3.44 (0.13)	3.05 (0.12)		2.89 (0.12)
Stop no-load	3.47 (0.13)	3.12 (0.12)		3.02 (0.13)
Double load	3.42 (0.14)	3.15 (0.15)		2.96 (0.17)
Double no-load	3.29 (0.12)	3.05 (0.13)		2.91 (0.15)
Experiment 3				
Rise	2.82 (0.17)	2.63 (0.18)		2.57 (0.19)
No-rise	2.79 (0.19)	2.61 (0.19)		2.55 (0.21)
Experiment 4				
Control load	3.81 (0.17)	3.37 (0.19)		3.00 (0.21)
Control no-load	3.91 (0.17)	4.47 (0.19)		3.00 (0.20)
Low-problem g. load	3.51 (0.26)	3.04 (0.25)		2.69 (0.28)
Low-problem g. no-load	3.63 (0.23)	3.26 (0.24)		2.95 (0.27)
High-problem g. load	4.21 (0.29)	4.17 (0.32)		3.88 (0.35)
High-problem g. no-load	4.22 (0.28)	4.04 (0.30)		3.99 (0.33)
	Part			
	1	2	3	4
Experiment 1				
Stop load	3.65 (0.12)	3.21 (0.13)	2.86 (0.13)	2.80 (0.15)
Stop no-load	3.57 (0.11)	3.22 (0.12)	3.07 (0.13)	2.97 (0.16)
Double load	3.28 (0.14)	3.08 (0.16)	3.26 (0.17)	3.08 (0.16)
Double no-load	3.23 (0.13)	2.92 (0.13)	3.02 (0.15)	3.15 (0.16)
Experiment 3				
Rise	2.86 (0.21)	2.67 (0.19)	2.54 (0.20)	2.64 (0.22)
No-rise	2.96 (0.22)	2.74 (0.20)	2.46 (0.19)	2.46 (0.20)
Experiment 4				
Control load	3.66 (0.14)	3.47 (0.14)	3.25 (0.14)	3.23 (0.14)
Control no-load	3.50 (0.14)	3.69 (0.14)	3.30 (0.13)	3.26 (0.13)
Low-problem g. load	3.64 (0.14)	2.48 (0.14)	2.80 (0.15)	2.44 (0.15)
Low-problem g. no-load	3.78 (0.14)	3.30 (0.15)	3.05 (0.17)	3.01 (0.15)
High-problem g. load	4.16 (0.18)	4.15 (0.19)	4.13 (0.19)	3.16 (0.20)
High-problem g. no-load	3.85 (0.17)	4.09 (0.19)	4.36 (0.18)	3.76 (0.19)
*Note.* g. = gambler.				

**Table 3 tbl3:** Results of the Analyses of Variance

Experiments and factors	*df*_1_	*df*_2_	*F*	*p*	Gen. η^2^
Experiment 1					
Stake (low, medium, high)	2	124	22.352	.000	.031
Load (no-signal vs. signal)	1	62	0.035	.851	.000
Group × Stake	2	124	0.330	.719	.000
Group × Load	1	62	4.245	.044	.001
Stake × Load	2	124	1.373	.258	.000
Group × Stake × Load	2	124	0.012	.988	.000
Part (1–4)	3	186	11.518	.000	.022
Group × Part	3	186	6.396	.000	.012
Part × Load	3	186	1.464	.223	.001
Group × Part × Load	3	186	1.853	.139	.001
Experiment 3					
Stake (low, medium, high)	2	62	3.962	.024	.010
Load (rise vs. no-rise)	1	31	0.137	.713	.000
Stake × Load	2	62	0.003	.996	.000
Part	3	93	3.715	.014	.019
Part × Load	3	93	1.433	.238	.002
Experiment 4					
Group (control, low-problem, high-problem)	2	69	3.688	.030	.088
Stake (low, medium, high)	2	138	45.020	.000	.042
Load (no-signal vs. signal)	1	69	4.037	.048	.001
Group × Stake	4	138	3.683	.007	.007
Group × Load	2	69	1.798	.173	.001
Stake × Load	2	138	0.849	.430	.000
Group × Stake × Load	4	138	2.785	.029	.001
Part	3	207	4.834	.003	.011
Group × Part	6	207	3.056	.007	.014
Part × Load	3	207	2.933	.035	.002
Group × Part × Load	6	207	1.027	.409	.001
*Note.* We analyzed betting scores using load, stake and part as within-subject variables and group as a between-subject variable. We ran separate analyses of variance (ANOVAs) for the Group × Load × Stake interaction and the Group × Load × Part interaction because there were insufficient trials for a full factorial analysis. To avoid redundancy, we only report effects of part for the second ANOVA. *df* = degrees of freedom; Gen. = generalized.					

**Table 4 tbl4:** Analyses of Variance for the Eye-Tracking Data in Experiment 1

Analyses and factors	*df*_1_	*df*_2_	*F*	*p*	Gen. η^2^
Region analysis					
Load	1	60	16.362	.000	.214
Group	1	60	0.599	.442	.010
Region	5	300	74.141	.000	.869
Load × Group	1	60	1.917	.171	.031
Load × Region	5	300	3.015	.018	.212
Region × Group	5	300	0.480	.790	.041
Load × Group × Region	5	300	0.189	.966	.017
Amount analysis					
Load	1	60	16.362	.000	.214
Group	1	60	0.599	.442	.010
Amount	5	300	3.086	.084	.409
Load × Group	1	60	1.917	.171	.031
Load × Amount	5	300	0.625	.432	.122
Amount × Group	5	300	0.863	.357	.150
Load × Group × Amount	5	300	1.403	.241	.214
*Note.* *df* = degrees of freedom; Gen. = generalized.					

**Table 5 tbl5:** Overview of the Betting Scores (Means, With Standard Deviations in Parentheses) for Experiments 2a and 2b

Experiments and conditions	Stake			Part		
	Low	Medium	High	1	2	3
Experiment 2a						
Accuracy	2.87 (0.21)	2.59 (0.19)	2.53 (0.18)	2.87 (0.21)	2.59 (0.19)	2.53 (0.18)
Speed	2.89 (0.20)	2.59 (0.20)	2.47 (0.18)	2.89 (0.20)	2.59 (0.20)	2.47 (0.18)
Experiment 2b						
Signal	3.04 (0.13)	2.76 (0.16)	2.65 (0.14)	3.00 (0.15)	2.82 (0.15)	2.64 (0.17)
No-signal	3.22 (0.14)	2.81 (0.13)	2.69 (0.13)	3.09 (0.15)	2.85 (0.14)	2.77 (0.17)

**Table 6 tbl6:** Results of the Analyses of Variance for Experiments 2a and 2b

Experiments and factors	*df*_1_	*df*_2_	*F*	*p*	Gen. η^2^
Experiment 2a					
Stake (low, medium, high)	2	62	10.396	.000	.022
Load (accuracy vs. speed)	1	31	0.057	.812	.000
Stake × Load	2	62	0.419	.659	.000
Part	2	62	0.577	.564	.001
Part × Load	2	62	0.559	.574	.000
Experiment 2b					
Stake (low, medium, high)	2	62	25.749	.000	.059
Load (signal vs. no-signal)	1	31	4.159	.050	.003
Stake × Load	2	62	2.416	.097	.002
Part	2	62	4.125	.021	.026
Part × Load	2	62	0.296	.746	.000
*Note.* *df* = degrees of freedom; Gen. = generalized.					

**Table 7 tbl7:** Gambler Characteristics in Experiment 4

Group	*n*	Average age (years)	Gender (f)	SOGS
Control	24	28	7	0.04
Low problem	24	28	8	1.60
High problem	24	35	12	9.10
*Note.* f = female; SOGS = South Oaks Gambling Screen.				

**Table 8 tbl8:** Betting Scores (Means, With Standard Deviations in Parentheses) for the Combined Analyses

Analyses and conditions	Stake			Part	
	Low	Medium	High	1	2
Combined analysis 1					
Stop load	3.15 (0.07)	2.77 (0.07)	2.51 (0.08)	2.97 (0.07)	2.65 (0.08)
Stop no-load	3.24 (0.07)	2.83 (0.07)	2.60 (0.07)	3.01 (0.07)	2.77 (0.07)
Double load	3.28 (0.08)	2.89 (0.08)	2.67 (0.08)	3.03 (0.08)	2.85 (0.09)
Double no-load	3.18 (0.08)	2.85 (0.07)	2.66 (0.08)	3.00 (0.07)	2.79 (0.08)
Combined analysis 2 (stop only)					
Load	3.43 (0.08)	3.07 (0.09)	2.78 (0.09)	3.25 (0.08)	2.94 (0.09)
No-load	3.50 (0.08)	3.13 (0.08)	2.88 (0.09)	3.29 (0.08)	3.06 (0.09)

**Table 9 tbl9:** Results of the Analyses of Variance for the Combined Analyses

Analyses and factors	*df*_1_	*df*_2_	*F*	*p*	Gen. η^2^
Combined analysis 1					
Group	1	214	0.266	.606	.001
Stake	2	428	154.892	.000	.047
Load	1	214	0.401	.527	.000
Part	1	214	33.014	.000	.011
Group × Stake	2	428	0.611	.543	.000
Group × Load	1	214	7.055	.008	.001
Stake × Load	2	428	1.424	.242	.000
Group × Part	1	214	1.047	.307	.000
Part × Load	1	214	0.373	.542	.000
Stake × Part	2	428	1.676	.188	.000
Group × Load × Stake	2	428	1.160	.314	.000
Group × Load × Part	1	214	2.671	.104	.000
Group × Stake × Part	2	428	0.439	.645	.000
Load × Stake × Part	2	428	0.300	.741	.000
Group × Load × Stake × Part	2	428	0.308	.735	.000
Combined analysis 2					
Stake	2	358	121.872	.000	.041
Load	1	179	7.055	.008	.001
Part	1	179	26.624	.000	.011
Load × Stake	2	358	0.967	.381	.000
Load × Part	1	179	4.305	.039	.000
Stake × Part	2	358	1.731	.178	.000
Load × Stake × Part	2	358	0.167	.846	.000
*Note.* *df* = degrees of freedom; Gen. = generalized.					

**Table 10 tbl10:** Results of the Analysis of Variance for Experiment 5

Factors	*df*_1_	*df*_2_	*F*	*p*	Gen. η^2^
Domain	1	31	0.992	.327	.019
Load	1	31	1.211	.280	.001
Domain × Load	1	31	2.655	.113	.004
*Note.* *df* = degrees of freedom; Gen. = generalized.					

## Footnotes

1 In this task, *risk-taking behavior* refers to preferring relatively high amounts with a low probability of winning (and, therefore, a low expected value) over low amounts with a high probability of winning. This is commonly seen as risk-taking behavior (and it is the behavior that pathological gamblers engage in). However, this is different from what some researchers have defined as risk taking—namely preferring an uncertain prospect (e.g., a 50% chance of winning £100 and a 50% chance of winning £50) over a certain prospect (100% chance of winning £75) with the same expected value.

2 Most individuals tend to be risk averse (Kahneman & Tversky, 1984), so we assume that the subjects in our experiment were also generally risk averse.

3 In the SCR analysis, there were more observations per cell than in the eye-movement analysis, in which we had to include region or amount. Therefore, in the initial SCR analysis, we also performed a Group × Load × Part analysis. This three-way interaction was not significant (*p* = .79). The main effect of part and the two-way Part × Group and Part × Load interactions were also nonsignificant (all *F*s < 1). Because of the slow nature of event-related changes in SCR levels, we could not assess the effect of stake on SCR levels.

4 We used Bayesian *t* tests to test whether load *decreased* gambling. Because the direction of the effect in the double-response group is opposite of that in the stop group, deducting no-load from load leads to a Bayesian factor lower than 0.33. The low Bayesian factor in the double-response group reflects the numerical *increase* in betting scores with load.

5 We thank an anonymous reviewer for suggesting this possible link between our manipulation and pop-up messages.

6 In the replication study of Verbruggen et al. (2012), there were 10 blocks, so the first “half” consisted of six blocks and the second “half” of four blocks.

7 The appetitive/aversive account does not directly predict differences in processing styles or changes in arousal (see Experiment 1).
