# Supplementary material for: How Does Response Inhibition Influence Decision Making When Gambling?
Source: J Exp Psychol Appl. 2015 Jan 5;21(1):15–36. doi: 10.1037/xap0000039 (PMC4353260; doi:10.1037/xap0000039)
Supplement: Supplementary file 1 [file xap-XAP-2014-1297-SupplementalMaterial.docx]

**Supplementary materials**

**EEG Bar task Experiment**

**Introduction**

In this experiment we combined the bar task with EEG to study the psychophysiological correlates of decision-making in load and no-load blocks. As mentioned in the Combined Analysis section of the main manuscript, we did not find an interaction between load and group. In this section, we report the full analysis of the behavioural data of this experiment. Because there was no statistically significant behavioural effect, we did not analyze the EEG results.

**Methods**

**Subjects.** 64 volunteers (45 female, mean age = 21 years) from the University of Exeter community participated for monetary compensation (£10), which was unrelated to performance. Written informed consent was obtained after the nature and possible consequences of the studies were explained. The study was conducted in accordance with the regulations laid out by the Exeter School of Psychology ethics committee.

**Apparatus, stimuli, and behavioural procedure**. Stimuli were presented on a 21-inch CRT monitor against a grey background. The task was run using PsychToolbox (Brainard et al. 1997). After the EEG setup, subjects completed the bar task, which was identical to one used in Experiment 1. Half of the subjects were allocated to the stop group, the other half to the double-response group.

**Results**

Betting scores are presented in Table S1. A complete overview of the analyses of variance is given in Table S2. Contrary to our hypothesis, subjects in the stop group tended to select *higher* amounts in load blocks (bet score = 2.67) than in no-load blocks (2.63), whereas subjects in the double-response group tended to select lower amounts in the load blocks (2.69) than in the no-load blocks (2.72). The interaction between Group and Load was not reliable (*p* = .500, B = .99).

An analysis of choice latencies in the gambling task showed that response latencies in the stop group were 102 ms longer in the load condition (93ms) than in the no load condition (-9). Response latencies in the double-group people were 42 ms longer in the load condition (4 ms) than in the no load condition (-38 ms). The interaction between Group and Load was significant, which supports the conclusion that the response slowing was more pronounced in the stop group than in the double group (Table S2).

**Table S1:** Overview of the descriptive for the EEG experiment.

|  | **Betting scores** | | | | | |
| --- | --- | --- | --- | --- | --- | --- |
|  | **Stake** | | | | | |
|  | **Low** | | **Medium** | | **High** | |
| Stop Load | 3.01(.11) | | 2.63(.12) | | 2.37(.15) | |
| Stop No-load | 2.93(.10) | | 2.58(.11) | | 2.39(.12) | |
| Double Load | 2.54(.15) | | 2.60(.14) | | 2.54(.15) | |
| Double No-load | 2.89(.14) | | 2.67(.14) | | 2.60(.15) | |
|  | **Part** | | | | | |
|  | **1** | **2** | | **3** | | **4** |
| Stop Load | 2.94(.12) | 2.72(.14) | | 2.55(.12) | | 2.51(.14) |
| Stop No-load | 2.83(.10) | 2.72(.11) | | 2.72(.12) | | 2.52(.12) |
| Double Load | 2.91(.16) | 2.68(.14) | | 2.55(.14) | | 2.63(.16) |
| Double No-load | 2.97(.15) | 2.72(.13) | | 2.67(.14) | | 2.52(.15) |

**Table S2:** Results of the Analysis of Variance

|  | ***Df1*** | ***Df2*** | ***F*** | ***p*** | ***Gen. eta^2^*** |
| --- | --- | --- | --- | --- | --- |
| Group | 1 | 62 | .044 | .834 | .001 |
| Load | 1 | 62 | .005 | .943 | .000 |
| Stake | 2 | 124 | 24.506 | .000 | .033 |
| Group by Load | 1 | 62 | .407 | .529 | .000 |
| Group by Stake | 2 | 124 | 1.883 | .156 | .003 |
| Stake by Load | 2 | 124 | 1.670 | .192 | .000 |
| Group by Stake by Block | 2 | 124 | .357 | .700 | .000 |
|  |  |  |  |  |  |
| Part | 1 | 186 | 9.615 | .000 | .019 |
| Group by Part | 3 | 186 | .215 | .886 | .000 |
| Part by Load | 3 | 186 | .296 | .828 | .000 |
| Group by Part by Load | 3 | 186 | 1.268 | .287 | .001 |

**Slot Machine Experiment**

**Introduction**

In this experiment we designed a slot machine game to examine whether the stop manipulation would have a similar effect on gambling behaviour in a different kind of gambling task. We introduced near misses in an attempt to make the game more engaging and increase gambling.

**Method**

**Subjects.** 48 volunteers (34 female, mean age = 20 years) from the University of Exeter community participated for monetary compensation (£5), which was unrelated to performance. Written informed consent was obtained after the nature and possible consequences of the studies were explained. The study was conducted in accordance with the regulations laid out by the Exeter School of Psychology ethics committee.


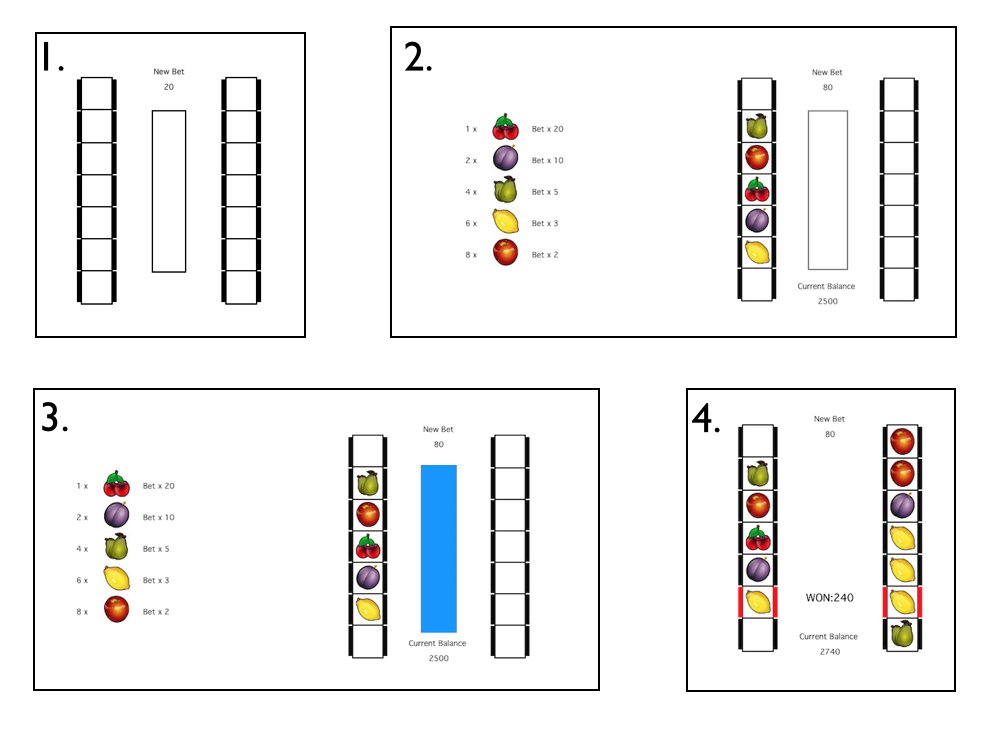


**Figure S1:** A typical trial in the slot machine task. First the amount subjects could bet was presented. After 1000ms, the response options, a reminder of the number of fruits, and the ‘payout’ associated with each fruit was presented. After 3.500 ms, the rectangle in the center would fill and subjects had to select one of the 5 fruits by pressing the corresponding key (see Procedure section). When a response was registered (in this example, the lemon was selected), the choice was confirmed by highlighting the selected fruit and the wheel on the right would spin for approximately 3000-4000 ms. When the wheel stopped, subjects would see if they had won or lost: they had won if the fruit on the right matched the selected fruit on the left.

**Apparatus, stimuli, and behavioural procedure**. Stimuli were presented on a 17-inch CRT monitor against a white background. The task was run using PsychToolbox (Brainard et al. 1997). Figure S1 shows a typical trial in the slot machine task.

On each trial the wager or current bet (5, 10, 20, 40, 80; i.e. the amount of points for which the subject would play) was displayed (Figure S1, Panel 1). The current bet remained visible throughout the trial.

After 1000 ms the other information was displayed (see Figure S1, Panel 2). Two wheels (the ‘choice’ wheel on the left and the ‘spinning’ wheel on the right) and a rectangular box (response box) were presented in the center of the screen. In addition we presented the 5 possible response options and their associated values. More specifically, subjects could choose one of 5 fruits. Each fruit occurred a certain number of times in the spinning wheel: there were 8 apples, 6 lemons, 4 pears, 2 plums, and 1 cherry. On a ‘win’ trial (i.e. when the fruit in the spinning wheel matched the selected fruit; see Panel 4 of Figure S1 and Figure S2), the bet was multiplied by a certain factor: for apples, the bet was multiplied by 2; for lemons, the bet was multiplied by 3; for pears, the bet was multiplied by 5; for plums, the bet was multiplied by 10; finally, for cherries, the bet was multiplied by 20. Table S3 summarizes the odds and payoff. The numbers and odds remained the same throughout the whole experiment.

**Table S3:** An overview of the number of fruits in the spinning wheel, the probability of winning for each fruit (=the number of a particular fruit divided by 21, which is the total number of fruits in the spinning wheel), the multiplication factor used to determine the number of points won, and the expected value of each option when the current bet or wager = 5 (note EV increases when the wager increases; for example, when the wager is 10, EV = 2xEV presented the last column).

|  | Number of fruits in the spinning wheel | P(win) | Bet multiplication | Expected value for wager = 5 |
| --- | --- | --- | --- | --- |
| Apple | 8 | 0.38 | 2 | 0.71 |
| Lemon | 6 | 0.29 | 3 | 0.71 |
| Pear | 4 | 0.19 | 5 | 0.71 |
| Plum | 2 | 0.10 | 10 | 0.24 |
| Cherry | 1 | 0.05 | 20 | 0.00 |

After 3500 ms the response box started filling up (Figure S1, Panel 3). When the response box was completely filled, the subject had to select a fruit on the choice wheel (similarly to the bars in the bar task), by pressing a button of the keyboard that was turned 90 degrees (with the space bar to the right). The subject was instructed to place their five fingers of the right hand on the response buttons (from bottom to top: “space bar”, “v”,”b”,”n” or ”m”), with the thumb on the space bar. This configuration was chosen so that the response buttons would be placed in a vertical position, similar to the betting options in each trial. In the example (Figure S1), if subjects wanted to select the lemon, they had to press the space bar with their thumb; if they wanted to select the pear, they had to press the ‘m’ key with their little finger.

When a response was registered, the right wheel would start spinning. It would eventually start slowing down until it stopped after approximately 3000-4000 ms. The subject won points if the fruit in the selected cell on the choice wheel matched the fruit in the corresponding cell in the spinning wheel (Panel 4, Figure S1). The amount won was the current bet multiplied by the factor of the selected fruit (see above). If the fruits didn’t match, subjects lost the amount indicated by the current bet. Any winnings or losses were deducted from the overall points displayed below the central box. The spinning of the wheel was pseudo-randomly controlled to ensure that p(win) roughly corresponded to the values in Table S3. Furthermore, 30 percent of the ‘loss’ trials were a ‘near miss’ trial (the chosen fruit appeared in the cell before or the cell after the ‘critical’ cell; see Figure S2, Panels B & C, respectively). The remaining loss trials were ‘far’ misses (i.e. the wheel would stop 3 or more cells away from what would have been a win; see Figure S2, Panel D). To create far misses, we grouped the fruits in the spinning wheel: there were 2 groups of 4 apples, 2 groups of 3 lemons, 2 groups of 2 pears, 2 groups of 1 plum, and 1 cherry. The order of groups was randomized across trials (with the restriction that groups of the same fruit never occurred after each other).


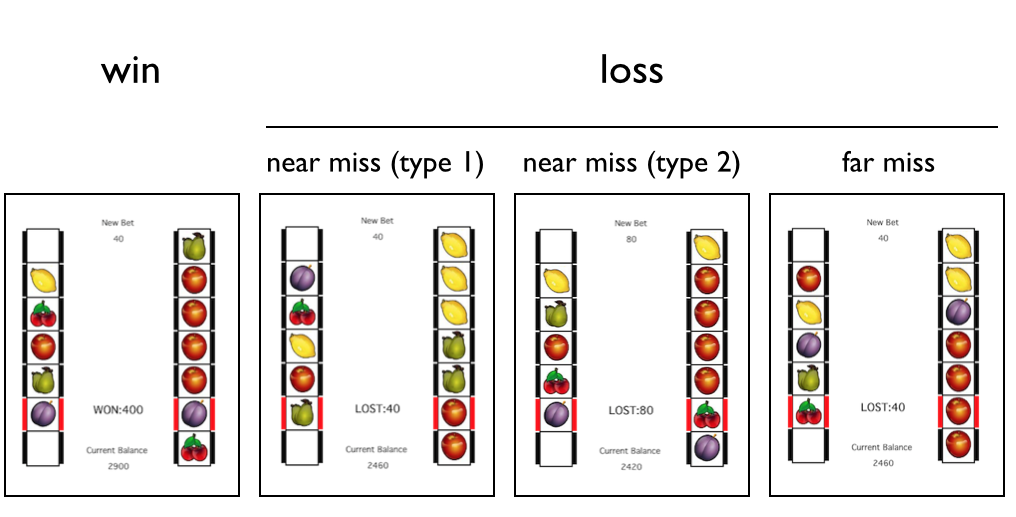


**Figure S2:** an overview of the four possible outcomes in the game.

Similarly to Experiment 1 of the main manuscript, subjects were randomly assigned to the Stop group (N = 24) or the Double response group (N = 24). The signal procedure was the same as in the bar task, apart from the following. On 25% of the trials in load blocks (signal trials), the fill colour would change from blue to black after a variable delay, instructing subjects in the double-group to press the response key twice and instructing subjects in the stop group to withhold their response.

The experimented consisted of 10 blocks of 20 trials. Half of the blocks were load blocks, and order of load and no-load blocks was counterbalanced. Before the experiment started the subject performed a short training phase in which we presented only the response box. Subjects practiced responding at the right moment (i.e. when the response box was fully filled) and stopping when a signal occurred (2 x 8 trials). This training phase was followed by a brief practice run of the actual gambling game (2 x 8 trials).

**Results**

Betting Score was calculated in a similar way as in the bar task: the most frequent fruit (i.e. apple) was 1 point, the least frequent fruit (i.e. the cherry) was 5 points.

**Table S4:** Betting scores in the slot-machine experiment

|  | **Wager** | | | | |
| --- | --- | --- | --- | --- | --- |
|  | **5** | **10** | **20** | **40** | **80** |
| Stop Load | 3.02(.15) | 2.48(.14) | 2.31(.12) | 2.09(.09) | 1.96(.10) |
| Stop No-load | 3.06(.14) | 2.78(.12) | 2.24(.10) | 2.13(.09) | 1.90(.09) |
| Double Load | 3.56(.14) | 2.95(.14) | 2.36(.11) | 2.27(.11) | 1.88(.13) |
| Double No-load | 3.58(.14) | 2.98(.12) | 2.48(.11) | 2.25(.11) | 1.91(.12) |
|  | **Part** | | | | |
|  | **1** | **2** | **3** | **4** | **5** |
| Stop Load | 2.46(.09) | 2.41(.09) | 2.32(.13) | 2.36(.14) | 2.29(.11) |
| Stop No-load | 2.44(.08) | 2.38(.09) | 2.38(.09) | 2.50(.13) | 2.57(.10) |
| Double Load | 2.45(.09) | 2.56(.12) | 2.72(.12) | 2.69(.11) | 2.57(.10) |
| Double No-load | 2.63(.08) | 2.78(.12) | 2.70(.11) | 2.50(.10) | 2.57(.11) |

*Betting scores.* A complete overview of betting scores and the analyses of variance is given in Tables S4-S5. There was a reliable Group by Load by Part interaction (p = .02; Table S5). Table S4 shows that in the later part of the experiment, subjects in the stop group tended to prefer fruits with a higher probability of winning in the load blocks than in the no-load blocks. The opposite pattern was observed in the double group as subjects tended to prefer fruits with a higher probability of winning in the later load blocks. Inspection of Table S4 shows that the effect of load was especially pronounced in the low bets (in particular the 10 point bet) in the Stop group. Such an effect is not observed in the double response group. The Group by Load by Bet was marginally significant (*p* = .074). This interaction indicates that stopping primarily influenced performance on trials in which the tendency to bet was highest.

**Table S5:** Results of the Analysis of Variance

|  | ***Df1*** | ***Df2*** | ***F*** | ***p*** | ***Gen. eta^2^*** |
| --- | --- | --- | --- | --- | --- |
| Group | 1 | 46 | 1.691 | .200 | .019 |
| Load | 1 | 46 | .996 | .323 | .001 |
| Bet | 4 | 184 | 42.388 | .000 | .266 |
| Group by Load | 1 | 46 | .0195 | .889 | .000 |
| Group by Bet | 4 | 184 | 1.663 | .160 | .014 |
| Load by Bet | 4 | 184 | 1.305 | .270 | .001 |
| Group by Load by Bet | 4 | 184 | 2.167 | .074 | .002 |
|  |  |  |  |  |  |
| Part | 4 | 184 | 0.195 | .941 | .001 |
| Group by Part | 4 | 184 | 0.734 | .569 | .004 |
| Part by Load | 4 | 184 | 0.541 | .706 | .001 |
| Group by Part by Load | 4 | 184 | 2.940 | .022 | .005 |
